# Effect of pachinko parlour openings and closings on neighbourhood income-generating crimes in Japan: 6.5 years of observations

**DOI:** 10.1186/s12889-024-19373-1

**Published:** 2024-07-16

**Authors:** Kenji Yokotani, Nobuhito Abe, Tetsuya Yamamoto, Masahiro Takamura, Hideyuki Takahashi

**Affiliations:** 1 Graduate School of Technology, Industrial and Social Sciences, Tokushima University, 1-1, Minamijosanjimacho, Tokushima City, Tokushima 770-0814 Japan; 2 Institute for the Future of Human Society, Kyoto University, Kyoto City, Japan; 3 Institutional Research Center, Fujita Health University, Toyoake City, Japan; 4 Graduate School of Engineering Science, Osaka University, Toyonaka City, Japan

**Keywords:** Electronic gambling machine, Daily income-generating offence rate, Convenience store, Geographic information, National longitudinal dataset, Japan, Pachinko parlour

## Abstract

**Background:**

Electronic gambling machines (EGMs) in gambling venues cause gambling-related harm and are a public health concern. This study focused on pachinko parlours as gambling venues and income-generating crimes as gambling-related harm. We aimed to verify whether income-generating crime rates increase in proximity to pachinko parlours and during the opening and post-closing periods of pachinko parlours relative to the pre-opening periods.

**Methods:**

We used crime records spanning 6.5 years, including data on the opening and closing days of pachinko parlours for 6.5 years. We also sampled the addresses of convenience stores, bowling alleys, and households with official land prices all over Japan. The dependent variable was the daily income-generating crime incidence rate. Areas within 0.5 km, 0.5–1 km, 1–5 km, and 5–10 km radii of the pachinko parlours were the independent variables. The pre-, opening-, and post-closing periods of the pachinko parlours were also independent variables. The covariates included the number of convenience stores and always open pachinko parlours near pachinko parlours. Data were analysed using an analysis of variance (ANOVA) and covariance (ANCOVA). We also used differences-in-differences analysis (DD) to reveal the increase in income-generating crime rates in neighbourhoods exposed to the opening or closing of pachinko parlours.

**Results:**

The daily income-generating crime incidence rate was significantly higher in areas within 0.5–1 km and 1–5 km radii of pachinko parlours than in those within 0.5 km and 5–10 km radii of them. The daily income-generating crime incidence rate was also significantly higher during the opening and post-closing periods than during the pre-opening period, even when controlling for the number of convenience stores and always open pachinko parlours. In particular, fraud crime rates increased with the opening and closing of pachinko parlours.

**Conclusions:**

The highest income-generating crime incidence rate was observed within a 0.5–1 km and 1–5 km radius of pachinko parlours. The opening of pachinko parlours also increased income-generating crime incidence rates, which increased after closing. Pachinko parlours are considered to be creating public harm because the corporate activities of these parlours make the youth in their neighbourhood perpetrators of fraud and older adults its victims. Future research should examine the current findings using official crime records.

**Supplementary Information:**

The online version contains supplementary material available at 10.1186/s12889-024-19373-1.

## Background

Gambling venues with electronic gambling machines (EGMs) cause gambling-related harm and are a public health concern [1]. Although EGMs offer leisure activities to people with lifelong disabilities [2], their excessive use is relevant to gambling disorders [3] and exploits savings more than necessary [4]. Moreover, areas near gambling venues are often characterised by poverty [5] because people who live near these venues often face bankruptcy from gambling activities [6]. These findings indicate the necessity of assessing and controlling the impact of gambling venues on neighbouring environment [7]. However, few studies have examined the association between gambling venues and their neighbouring environments using individual criminal cases that are not equalised by groups [6, 8, 9] and previous findings are limited to Western countries [10]. To address these issues, the current study focused on pachinko parlours in Japan because Japan has 56% of the world’s EGMs [11], most of which are located in these parlours [12]. We also used national longitudinal individual crime case records spanning 6.5 years to clarify the relationship between the opening/closing of pachinko parlours and the incidence of crime, which is a form of gambling-related harm. Note that the crime data utilised in this study were collected by a private company and do not ensure representativeness; thus, caution is required when interpreting the results.

### Theoretical framework

Our theoretical framework includes the regional exposure theory [13] and rational choice perspective [14]. According to the regional exposure theory, people who reside physically close to gambling venues are highly likely to develop gambling disorders because of their repeated exposure to gambling behaviours [15]. Residents living near gambling venues with EGMs are more likely to use these machines [16]. Residents of areas with a high concentration of gambling venues also experience high bankruptcy rates [6]. Furthermore, high concentrations of these venues have been positively correlated with a high prevalence of gambling disorder [17], particularly among male gamblers [9]. These findings suggest that physical proximity to gambling venues is associated with a high incidence of gambling disorders. Moreover, once a person is exposed to gambling activity in the vicinity of a gambling venue, the subsequent closure of the venue does not reduce gambling disorders [18, 19]. These findings suggest that physical proximity to gambling venues is associated with a higher incidence of persistent, long-term gambling disorders.

People with gambling disorder are also more likely to commit income-generating crimes, which comprise gambling-related harm [20], than those with no disorder, because the former are impoverished due to gambling but want money to gamble [21]. Several studies have highlighted the association between gambling disorders and income-generating crime [22]. Their craving for gambling is a factor in their propensity to engage in income-generating crimes [23]. Committing income-generating crimes is considered a diagnostic criterion for gambling disorder as well [24]. These findings indicate a high risk of income-generating crimes among people with gambling disorders.

From the rational choice perspective, income-generating perpetrators [14] tend to commit crimes at short distances from their homes, schools, or workplaces [25–27]. This is because such places are familiar, allowing them to plan multiple escape routes in advance or change them spontaneously, making them less likely to be arrested after committing a crime [25]. However, it is harder to commit crimes too close to the home, school, or workplace. Being too close increases the risk of being identified by witnesses who may recognise their home or daily commute, thereby increasing the likelihood of a later arrest [26]. Robberies are more likely to occur approximately 2.57 km away from the perpetrator’s home [25]. Therefore, it can be said that income-generating perpetrators with gambling disorder are most likely to commit crimes at a distance slightly away from the pachinko parlour.

Furthermore, this rational choice perspective [14] can predict that income-generating crime rates increase during the post-closing period of pachinko parlours. When pachinko parlours are operational, potential income-generating perpetrators with gambling disorders visit them frequently, making them less likely to commit crimes in the vicinity of the parlour for fear of being unmasked. However, when the parlour closes, they will no longer visit it; thus, the risk of being exposed will decrease, but they will remain familiar with the parlour. Consequently, it is easier for them to commit an income-generating crime in the vicinity of a closed pachinko parlour. In fact, perpetrators are more likely to commit crimes in places where they have lived in the past [27]. This is because, while they know the place because they live there and can easily prepare various escape routes, they do not currently live there and therefore have a low risk of being unmasked [27].

**Fig. 1:**
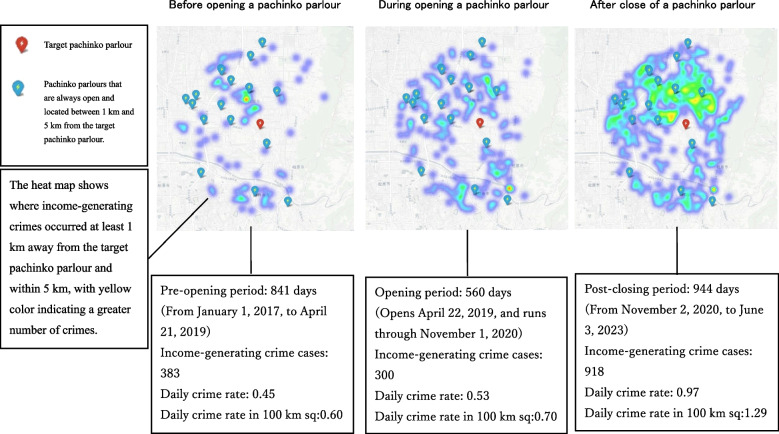
Calculation of the daily income-generating crime rate within 1 km to 5 km radius of a pachinko parlour

The studies described above naturally lead to the view that the physical proximity and opening/post-closing periods of gambling venues with EGMs facilitate the occurrence of gambling disorders and increase income-generating crimes. In fact, a study conducted at one gambling venue found an increase in the neighbourhood’s income-generating crime 1 year after the venue opened [8]. Further, many studies using year- and group-level datasets indicate the association between proximity to gambling venues and income-generating crime [28, 29]. However, only a few studies have supported this association by using day and individual-crime cases [6]. Hence, we examined the association at the daily and individual levels of datasets by combining the longitudinal and latitudinal data of criminal case records with the opening and closing dates of gambling venues (Fig. 1). To clarify this association, this study examined more than 10,000 gambling venues across Japan for 6.5 years.

### Current situation of pachinko parlours in Japan

Pachinko, or Pachislot, involves popular pinball EGMs in Japan, in which players launch small steel balls onto the playing field to capture them in certain patterns or slots for prizes. Although pachinko is officially meant for leisure [30], in reality, it is meant for gambling, and many pachinko parlours have pachinko or pachislot EGMs [9]. As of 2021, the market size of pachinko in Japan is 14.6 trillion Japanese yen per year [30]. The total Japanese gambling market is worth 22.9 trillion yen [31] per year, of which 63% can be considered the pachinko market. However, the pachinko market has been gradually shrinking. In fact, the number of pachinko parlours peaked in 1995 with 18,244 establishments, but by 2022, this number had dropped to 7,665 [12]. Similarly, the number of people visiting pachinko parlours within a year decreased from 29 million in 1995 to 7.7 million in 2022 [12].

There were two main reasons for this decline. The first was repeated revisions to gaming laws since 1990 [32]. These amendments frequently restricted the odds of winning and the number of balls that could be won by pachinko EGMs [33]. Owing to these legal changes, old pachinko EGMs became illegal, necessitating the removal of old EGMs and the introduction of new ones. In addition, parlours with old EGMs had to stop operating. Consequently, the investment costs for pachinko parlours increased, leading to more than ten parlours going bankrupt each year [34]. Moreover, these changes reduced the likelihood of players winning and diminished the appeal of playing pachinko. Indeed, a nationwide survey of people who used to play pachinko but no longer did so revealed that over 70% stopped playing pachinko because it became harder to win and less enjoyable [34]. In other words, repeated amendments to gaming laws have contributed to a decrease in both pachinko parlours and players.

The second reason was the spread of COVID-19. Actions to reduce the risk of COVID-19 were recommended throughout Japan. Pachinko parlours, which are enclosed spaces with players in close proximity, were considered high risk, leading to government-led closures and suspensions of many parlours [35]. Consequently, the number of pachinko players decreased by approximately 1.8 million from 2019 to 2020 [36]. Thus, the government’s response to the COVID-19 pandemic reduced the number of pachinko players.

Given the context of a shrinking market [12, 34, 36], the environment in which pachinko parlours close every year is suitable for examining trends in the rate of income-generating crimes before and after their closure. Furthermore, Japan has a large number of people with gambling disorders [37] and gambling-related harm [38]. In particular, Japanese men living in areas with pachinko parlours located within 1.5 km of their houses are highly likely to develop gambling disorders [9]. These findings are also suitable for examining the proximity effects of pachinko parlours on income-generating crime rates.

Our study also used the number of convenience stores near pachinko parlours. This is because the number of convenience stores in a given neighbourhood is associated with the population density and financial wealth of the area [39]. This population density and financial wealth are known to be positively correlated with income-generating crime rates [40, 41]; thus, we controlled for these effects. Furthermore, we controlled for the number of always open pachinko parlours near pachinko parlours. There are often competing pachinko parlours in the neighbourhood of one pachinko parlour [9], and the influence of these competitors must be controlled when predicting income-generating crimes. Bowling alleys were sampled for comparison with pachinko parlours. Japanese bowling alleys can be considered quasi-gambling venues because they are equipped with video game arcades [42], and their frequent use is relevant to gambling disorders [43, 44]. In this study, traffic crimes were also sampled for comparison with income-generating crimes [45]. Traffic crimes are associated with impulsivity among people with gambling disorders, but the association between traffic crimes and gambling disorders is weaker than that between income-generating crime and gambling disorder [46].

### Hypotheses

Our study proposed two main hypotheses: First, the daily income-generating crime rate increases in proximity to pachinko parlours but decreases when pachinko parlours are too close. Based on previous studies [6, 8, 9], this hypothesis was tested at 0.5 km, 0.5–1 km, 1–5 km, and 5–10 km radii of the pachinko parlours. The first main hypothesis was tested using two sub-hypotheses. Hypothesis 1a: The daily income-generating crime rates at 1–5 km radii of pachinko parlours are higher than those at 5–10 km radii of pachinko parlours. Hypothesis 1b: Daily income-generating crime rates at 0.5 km radii of pachinko parlours are lower than those at 0.5–1 km radii of pachinko parlours. In other words, the daily income-generating crime rate is expected to be in a mountain shape with 0.5–1 km and 1–5 km at the top in a radius of 0.5 km, 0.5–1 km, 1–5 km, and 5–10 km from the pachinko parlours.

**Fig. 2:**
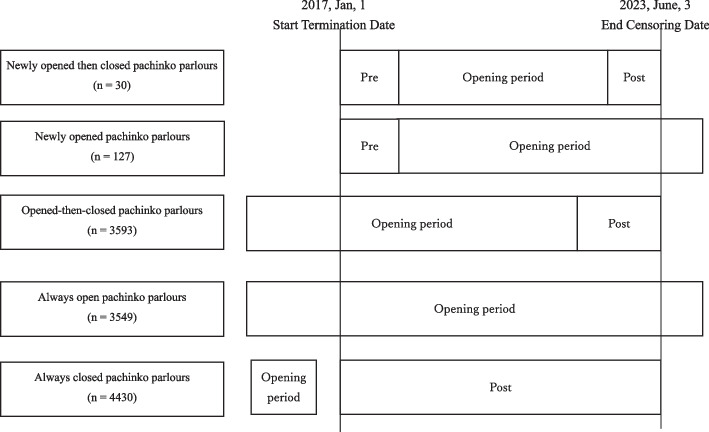
Five classes of pachinko parlours

Second, even after controlling for the number of pachinko parlours and convenience stores near them, the daily income-generating crime rate is higher during the opening and post-closing periods than during the pre-opening period. The second main hypothesis was tested using four sub-hypotheses. We focused on 30 newly opened then closed pachinko parlours (Fig. 2), which opened after 2 January 2017 and completely closed before 2 June 2023. As the sampling periods started from 1 January 2017 and ended on 3 June 2023, these parlours must have a pre-opening period, that is, the period prior to their opening, and a post-closing period, that is, the period after which the pachinko parlour completely closed. Hypothesis 2a: The daily income-generating crime rate in the opening and post-closing periods is higher than that in the pre-opening period among newly opened then closed pachinko parlours.

Next, we examined how the opening of pachinko parlours increased the daily income-generating crime rate in the neighbourhood. We compared 127 newly opened pachinko parlours (Fig. 2) with 552 bowling alleys. The former neighbourhood was exposed to the opening of a pachinko parlour, whereas the latter was not. Hypothesis 2b: Local residents of newly opened pachinko parlours will experience an increase in the daily income-generating crime rate during the opening period, but not in bowling alleys. Similarly, we examined how the closing of pachinko parlours increased the daily income-generating crime rate in the neighbourhood. We compared 3,593 open-then-closed pachinko parlours (Fig. 2) with 3,549 always open pachinko parlours (Fig. 2). The former neighbourhood was exposed to the closing of a pachinko parlour, whereas the latter was not. Hypothesis 2c: Open-then-closed pachinko parlours will experience an increase in the daily income-generating crime rate during the post-closing period, but not the always open pachinko parlours.

Finally, we compared the daily income-generating crime rates among 3,549 always open pachinko parlours, 3,593 open-then-closed pachinko parlours, and 4,430 always closed pachinko parlours (Fig. 2). Since closing pachinko parlours could increase the daily income-generating crime rate [25–27], the rate would be higher in 3,549 open pachinko parlours, 3,593 open-then-closed pachinko parlours, and 4,430 always-closed pachinko parlours (Hypothesis 2d).

These hypotheses were also tested by replacing income-generating crimes with traffic crimes, showing that they were substantiated only for income-generating crimes. Hypotheses 2b and 2c were tested again for fraud, robbery, purse snatching, and theft among the income-generating crimes to determine which crimes were most likely to occur after exposure to the opening/closing of pachinko parlours.

## Methods

### Study design and settings

We conducted observational studies using five nationwide datasets from Japan. The gambling dataset included 11,727 pachinko parlours. The crime dataset included 245,165 income-generating crimes and 58,387 traffic crimes. The sample period for these data was from 1 January 2017 to 3 June 2023. Data collected before and after this period were excluded from analysis. We also sampled 58,648 addresses from convenience stores [47], 552 addresses from bowling alleys [48], and 25,993 addresses from households with official land price appraisals [49].

### Datasets

**Fig. 3:**
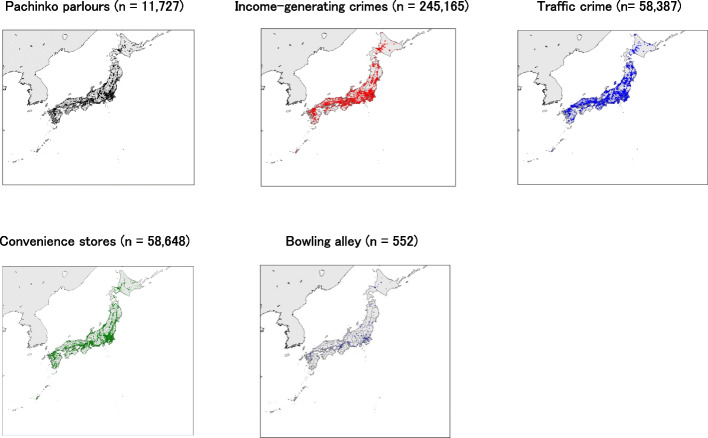
Geographic distribution of pachinko parlours, income-generating crimes, convenience stores, and bowling alleys in Japan. Notes. Black dots indicate the addresses of the pachinko parlours. Red dots indicate the locations of income-generating crimes. Blue dots indicate the locations of traffic crimes. Green dots indicate the addresses of convenience stores. Dark blue dots indicate the addresses of the bowling alleys

We used the Koko-Pachi website dataset to obtain the addresses and opening/closing dates of pachinko parlours in Japan [50]. The information in the dataset was based on publicly available pachinko parlours’ flyers or web pages, which guaranteed the reliability of the data. It recorded the opening and closing dates of pachinko parlours from 2005 to 2023. Based on these data, each pachinko parlour’s opening and closing dates from 1 January 2017 to 3 June 2023 were examined, and this period was aligned with the crime data sampling period. Pachinko parlours were identified by their addresses rather than store names. In other words, even when the pachinko parlours had different store names, they were considered a single parlour if they had the same address. Accordingly, 11,727 pachinko parlours were identified in this study. They included pachinko parlours that opened or closed before 1 January 2017. Furthermore, this number corresponded to 79% of the 14,805 officially registered pachinko parlours with EGMs in Japan [51]. The remaining 21% of the pachinko parlours reported their operations to the police but did not disclose their store information. These pachinko parlours may be unable to operate because of a lack of funds [34]. The addresses were converted into longitudes and latitudes using the Application Programming Interface (API) provided by the Geospatial Information Authority of Japan [52], as shown in Fig. 3. Most of the pachinko parlours in Japan were located in urban areas.

The pachinko parlours were divided into five classes (Fig. 2). First, we identified 30 newly open-then-closed pachinko parlours (Fig. 2). These pachinko parlours opened after 2 January 2017 and completely closed before 2 June 2023. The opening periods of these pachinko parlours were thus limited to 2 January 2017 to 2 June 2023. As the sampling period started on 1 January 2017, these parlours must have had a pre-opening period, that is, the period prior to the opening of the pachinko parlours. Similarly, since the sampling period ended on 3 June 2023, they also had a post-closing period, that is, the period after which the pachinko parlours completely closed.

Second, we identified 127 newly opened pachinko parlours (Fig. 2). These pachinko parlours opened after 2 January 2017 but never closed before 3 June 2023. According to the sampling periods between 1 January 2017 and 3 June 2023, these pachinko parlours had pre-opening and opening periods but did not have post-closing periods.

Third, we identified 3,593 open-then-closed pachinko parlours (Fig. 2). These parlours opened before 1 January 2017 and were completely closed before 2 June 2023. In contrast to the newly opened pachinko parlours, these parlours did not have a pre-opening period but had opening and post-closing periods.

Fourth, we identified 3,549 always open pachinko parlours (Fig. 2). These pachinko parlours opened before 1 January 2017 and never closed before 3 June 2023. These pachinko parlours only had open periods.

Fifth, we identified 4,430 always closed pachinko parlours (Fig. 2). These parlours were completely closed before 1 January 2017. They had only post-closing periods.

To obtain data on the date and location of criminal acts in Japan, we also accessed publicly available criminal act information provided by Gaccom Safety Navi [53]. To ensure data reliability, we considered only the crimes officially reported by the police or government. We identified the type, date, and location of crime occurrences from publicly available criminal act information (Additional file 1). To extract data from the text, ja-timex 0.2.7. was used [54]. The API of the Geospatial Information Authority of Japan [52] was used to convert the locations into longitudes and latitudes. Publicly available criminal act information has been published since 1999 based on public agency announcements, but this announcement has become widely available only since 2017 (Additional file 2). The reason for this is that Gaccom started its information service related to public safety in December 2016 [53]; since this service commenced only from December 2016, there is a significant lack of data prior to this date. However, since the service started in January 2017, the data from that point onwards are much more comprehensive. Therefore, this study used data from 1 January 2017 to 3 June 2023 (Additional file 2).

Based on the crime type of publicly available criminal act information (Additional file 1), crimes labelled ‘Robbery/Threatening’ (*n* = 7,941), ‘Purse snatching’ (*n* = 6,606), ‘Theft’ (*n* = 51,687), and ‘Fraud/False Pretence’ (*n* = 191,019) were identified as income-generating crimes. Several labels overlapped with one income-generating crime case; therefore, the sum of each label’s crimes was greater than the total number of income-generating crimes (*n* = 245,165).

Fraud in Japan is considered an organised crime, and fraud perpetrators are divided into specific roles [55]: the mastermind who directs the fraud overall, the collector who receives the money directly from the victims, the withdrawer who uses the victims’ cash cards to withdraw the fraudulently obtained money, and the watcher who monitors the collectors and withdrawers to prevent them from running off with the money. Among those arrested for fraud, 72.2% were collectors, withdrawers, or watchers and only 1.7% were masterminds [56]. These data indicate that arrested perpetrators in Japanese fraudulent cases are physically close to the victims and handle money directly. Therefore, fraud was categorised as an income-generating crime in this study.

**Fig. 4:**
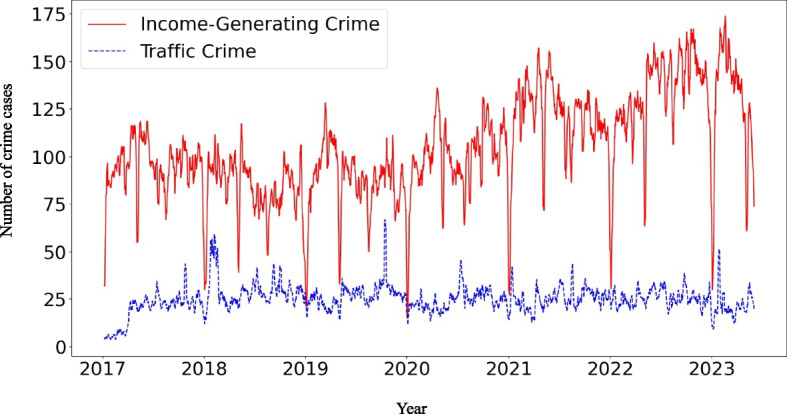
Trends in income-generating and traffic crime over time. Notes. Trends over time for income-generating and traffic crimes are shown in the daily crime data as a 7-day moving average

Figure 3 depicts the locations of 245,165 income-generating crimes. Although such crimes occur throughout Japan, most are concentrated in urban areas. However, these numbers represent only a portion of the total crimes occurring in Japan, with 381,769 thefts per year [57], compared to the 51,687 thefts in this study over 6.5 years. Figure 4 also shows the trends in income-generating crime over the years. Income-generating crime is likely to decrease during the year-end and New Year’s holidays.

Similar to income-generating crimes, crimes labelled ‘Traffic’ in the publicly available criminal act information (Additional file 1) were identified as traffic crimes (*n* = 58,387). Although the Gaccom Safety Navi has 31 labels, such as ‘violence’ [53], we used only income-generating and traffic labels. Figure 3 depicts the locations of 58,387 traffic crimes. Although such crimes occur throughout Japan, most are concentrated in urban areas. Figure 4 shows the trends in traffic crime over time.

We also sampled 58,648 convenience store addresses [47], 552 bowling alley addresses [48], and 25,993 household land addresses using official land value appraisals [49]. Based on their addresses, we collected their longitudes and latitudes using the API provided by the Geospatial Information Authority of Japan [52]. The location of the convenience store and bowling alley is shown on the map. Figure 3 shows that convenience stores and bowling alleys are concentrated in urban areas.

### Independent variables

Ranges within 0.5 km, 0.5–1 km, 1–5 km, and 5–10 km radii of the pachinko parlours were independent variables. The area of the range must be adjusted when comparing crime rates across four ranges. Therefore, an adjustment parameter was added to convert the area of these ranges to 100 km square (Additional file 3).

The pre-opening, opening, and post-closing periods of the pachinko parlours were used as independent variables. However, several sampling periods exceeded the required sampling period. If the period began before 1 January 2017, it was terminated on 1 January 2017 (Fig. 2). Similarly, if the period ended after 3 June 2023, it was terminated on 3 June 2023 (Fig. 2).

### Primary outcome

The incidence rate of income-generating crimes per day in the vicinity of pachinko parlours was calculated as follows. First: all the income-generating crimes that occurred within a 1–5 km radius of pachinko parlours were considered based on their longitudes and latitudes (Fig. 1). All income-generating crimes were assigned dates. Hence, based on these data, we identified the income-generating crimes that occurred during the pre-opening, opening, and post-closing periods and counted the number of income-generating crimes (Fig. 1). This number was divided by the period length (in days) in the three periods to estimate the incidence rate of income-generating crimes per day. This incidence rate was multiplied by 1.33 to adjust a circular area with a radius of 1–5 km into 100 km square (Additional file 3). Using the aforementioned method, we calculated the incidence rate of income-generating crimes per day in the suburbs within radii of 0.5 km, 0.5–1 km, and 5–10 km from pachinko parlours (Additional file 3). Notably, the wide area conditions did not include near area conditions. In other words, income-generating crimes occurring within a 5–10 km radius of pachinko parlours never included income-generating crimes occurring within 0.5 km, 0.5–1 km, and 1–5 km radii of these parlours (Additional file 3).

### Control variables

The number of convenience stores near pachinko parlours was used as a control variable because accessibility to food stores can be an index of wealth [39]. First, the number of convenience stores near the land with a land price appraisal was sampled, and the correlations between the number of convenience stores and land prices were examined (Additional file 4). The results showed that land price had a high correlation (*r* = 0.83) with the number of convenience stores in a 5 km radius around the land (Additional file 4). Therefore, the number of convenience stores within a 5 km radius of pachinko parlours was used as a control variable in this study.

The number of always open pachinko parlours near one pachinko parlour was used as the control variable. The number of always open pachinko parlours near pachinko parlours was estimated according to the 0.5 km, 0.5–1 km, 1–5 km, and 5–10 km radii of pachinko parlours (Fig. 1), because the income-generating crime rates were also calculated according to these radii (Additional file 3).

The year in which an income-generating crime occurred was also included as a control variable because the income-generating crime rate may change according to the year [58]. However, the year in which the crime occurred coincided with the pre-opening, opening, and post-closing periods of the pachinko parlours, making it difficult to remove the effect. Thus, when the year control variable was included, the independent variable for the period was excluded.

### Control (no exposure) group

To examine whether the daily income-generating crime rates increased in areas exposed to the opening of pachinko parlours, 552 bowling alleys not exposed to the opening were used as the control group. Bowling alleys in Japan are equipped with video game arcades, which can be considered quasi-gambling venues [42–44]; hence, bowling alleys with video game arcades were also regarded as quasi-gambling venues. The period before and after the opening of 127 newly opened pachinko parlours was randomly assigned to 552 bowling alleys. These 127 pachinko parlours were used as experimental groups exposed to the openings.

**Fig. 5:**
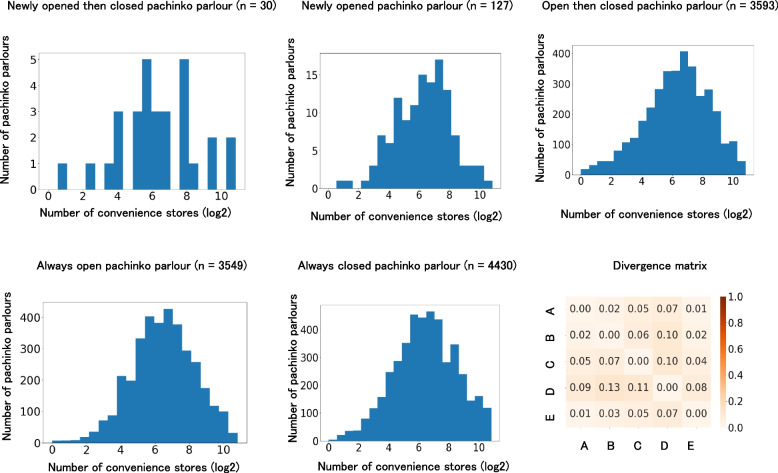
Comparison of locations of pachinko parlour among five business classes. Notes. The number of convenience stores was log-transformed to a base number of two after adding 1. **A** Always closed pachinko parlour, **B** Always open pachinko parlour, **C** Newly opened pachinko parlour, **D** Newly opened then closed pachinko parlour, **E** Opened then closed pachinko parlour

Moreover, to examine whether daily income-generating crime rates increased in areas exposed to the closing of pachinko parlours, 3,549 always open pachinko parlours that were not exposed to the closing of pachinko parlours were used for comparison (Fig. 2). There was little difference in location between the two types of pachinko parlours (Fig. 5). The period before and after the closing of 3,593 open-then-closed pachinko parlours was randomly assigned to 3,549 always open pachinko parlours. These 3,593 pachinko parlours were used in the experimental group exposed to the closing.

### Statistics

To test Hypotheses 1a and 1b, a two-way ANOVA (Eq. 1) was performed to investigate the effects of the four radii (0.5 km, 0.5–1 km, 1–5 km, and 5–10 km radii of pachinko parlours) and five pachinko parlours (Fig. 2) on the outcome variables (daily income-generating crime rate per 100 km square). Tukey’s honest significant difference test was conducted [59]. In Eq. 1, C is the crime rate, u is the intercept, d is the distance from the pachinko parlour, P is the class of the pachinko parlour, i is the individual address of the parlour, h is the distance of the parlour (Additional file 3), and j represents the five classes of the parlour (Fig. 2).

1 $${C}_{ihj}=u+{d}_{h}+{P}_{j}+{\varepsilon }_{ihj}$$

To test Hypothesis 2a, a two-way analysis of covariance (ANCOVA; Eq. 2) was performed to investigate the effect of three periods (pre-opening, opening, and post-closing periods) and four radii (0.5 km, 0.5–1 km, 1–5 km, 5–10 km radii of pachinko parlours) on the outcome variables (daily income-generating crime rate per 100 km square) with control variables (number of convenience stores and always open pachinko parlours near pachinko parlours). In Eq. 2, f is the period of pachinko parlour, S is the log-transformed number of convenient stores in the neighbourhood, A is the log-transformed number of always open pachinko parlours in the neighbourhood, β1 and β2 are coefficients, and k is three periods of the parlours. The other variables in Eq. 2 are the same as those in Eq. 1:

2 $${C}_{ihk}=u+{d}_{h}+{f}_{k}+{\beta }_{1}{S}_{i}+{\beta }_{2}{A}_{ih}+{\varepsilon }_{ihj}$$

To test hypotheses 2b and 2c, we used DD [60]. Based on a previous study [61], we used Eq. 3. In Eq. 3, a is the time-invariant characteristic of the group, b is the group-invariant characteristic of time, and D is the interaction effect between the group and time. g is a binary variable for the group, where 0 indicates that the group is not exposed and 1 indicates that the group is exposed. t is a binary variable for time, where 0 indicates before the exposure and 1 indicates after the exposure. The other variables in Eq. 3 are the same as those in Eqs. 1 and 2:

3 $${C}_{igt}={a}_{g}+{b}_{t}+{D}_{gt}+{\beta }_{1}{S}_{i}+{\beta }_{2}{A}_{ih}+{\varepsilon }_{igt}$$

When examining the exposure effect of the opening of pachinko parlours, newly opened pachinko parlours (Fig. 2) represented the exposed experimental group, and bowling alleys represented the unexposed control group. The period before exposure is referred to as the pre-opening period, and the period after exposure is referred to as the during-opening period. Similarly, when examining the exposure effect of the closing of pachinko parlours, open-then-closed pachinko parlours (Fig. 2) represent the exposed experimental group, whereas always open pachinko parlours represent the unexposed control group. The period before exposure is referred to as the during-opening period, and the period after exposure is referred to as the post-closing period.

In the current study, Differences in Differences (DD) implies the following: (the post-exposure mean of the exposed group – the pre-exposure mean of the exposed group) – (the post-exposure mean of the unexposed group – the pre-exposure mean of the unexposed group). According to the previous study [62], the effect size of DD is equivalent to Cohen’s d_av_: 2DD / (SD_exp_diff_ + SD_unexp_diff_), where SD_exp_diff_ is the standard deviation of the differences between the post-exposure values of exposed group and the pre-exposure values of exposed group and SD_unexp_diff_ is the standard deviation of the differences between the post-exposure values of the unexposed group and the pre-exposure values of the unexposed group. Furthermore, interaction effect is equivalent to the coefficient of ‘Treatment*Post-exposure’ in the Eq. (3) that controls for the number of convenience stores and always open pachinko parlours near a pachinko parlour.

To test Hypothesis 2d, a two-way ANCOVA (Eq. 4) was performed to investigate the effects of the three classes of pachinko parlours (always closed, always open, opened-then-closed) and the four radii (0.5 km, 0.5–1 km, 1–5 km, 5–10 km radii of pachinko parlours) on outcome variables (daily income-generating crime rate per 100 km square) with control variables (years when the income-generating crime occurred and the number of convenience stores and always open pachinko parlours near pachinko parlours). Tukey’s honest significant difference test was conducted [59]. In Eq. 4, Y is the year when the crime occurred, β3 is the coefficient, and l is 7 years (from 2017 to 2023). The other variables in Eq. 4 are the same as those in Eqs. 1, 2, and 3.

4 $${C}_{ihjl}=u+{d}_{h}+{P}_{j}+{\beta }_{1}{S}_{i}+{\beta }_{2}{A}_{ih}+{{\beta }_{3}Y}_{l}+{\varepsilon }_{ihjl}$$

Moreover, the Jensen-Shannon (JS) divergence was used to estimate the differences between two distributions because JS divergence can be used as an index of the distance between two distributions [63].

### Ethical considerations

This study was approved by the ethics committee of the Graduate School of Technology, Industrial and Social Sciences, Tokushima University on 9 August 2023 (registration number: 293). Because this study used publicly available data on the web, informed consent for research use was obtained only from those who made the data available to the public.

## Results

### Distributions of pachinko parlour location

Before testing our hypotheses, we conducted descriptive analyses of the economic features of each pachinko parlour class. The number of convenience stores within 5 km of the pachinko parlour was used to examine the economic features of its location. Figure 5 shows that all classes of pachinko parlours had approximately 64–256 or 2^6^–2^8^ convenience stores within a 5 km radius of them. This unimodal distribution is a general trend, as similar trends have been observed in the national land price survey data (Additional file 4).

Furthermore, a comparison of location distribution among the five classes showed that the JS divergence score for each class was less than 0.13 (Fig. 5), indicating that there is a small difference in pachinko parlour location in terms of the number of nearby convenience stores regardless of their class. These results indicate that pachinko parlours in Japan are evenly distributed across all areas and not unevenly distributed in poor neighbourhoods, as is the case with casinos in western countries [5, 64]. Nevertheless, the number of convenience stores near a pachinko parlour was positively correlated with daily income-generating offence rates, such as the number of always open pachinko parlours near a pachinko parlour (Additional file 5). Thus, these numbers need to be controlled when we compare different classes of pachinko parlours.

### Comparison of daily income-generating crime rate at 0.5 km, 0.5–1 km, 1–5 km, and 5–10 km radii from pachinko parlours: distance effects

**Fig. 6:**
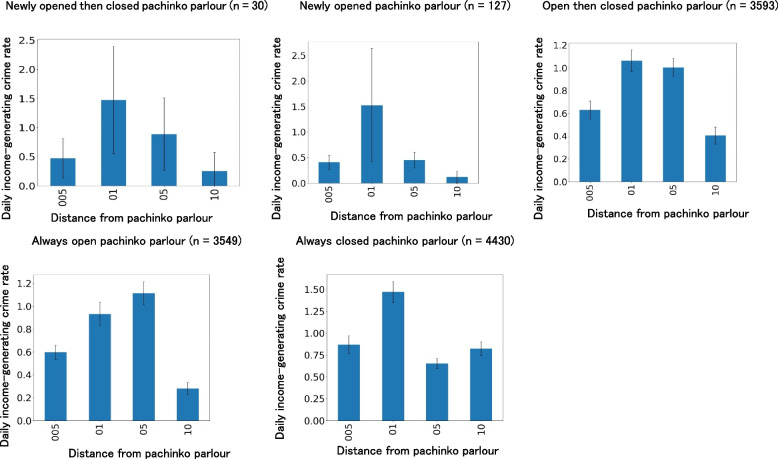
Comparison of daily income-generating crime rates within 0.5, 0.5 km to 1 km, 1 km to 5 km, and 5 km to 10 km of pachinko parlours in Japan. Notes. All areas with daily income-generating crime rates were adjusted to 100 km sq. The 005 horizontal line indicates the distance within 0.5 km of the pachinko parlours. The 01, 05, and 10 of the line indicate the distance within 0.5 km to 1 km, 1 km to 5 km, and 5 km to 10 km of pachinko parlours, respectively. The black bars indicate 95% confidence intervals. The effects of distance on daily income-generating crime rates were significant (*F* = 120.91, *df1* = 3, *df2* = 46,908, *p* < .001). The effects of pachinko parlours on daily income-generating crime rates were also significant (*F* = 15.43, *df1* = 3, *df2* = 46,908, *p* < .001). Multiple comparisons showed that the crime rate was significantly highest in the 0.5 km to 1 km range, followed by a significantly higher crime rate in the 1 km to 5 km range, then a significantly higher crime rate in the 0.5 km range, and the lowest crime rate in the 5 km to 10 km range; The crime rates within 0.5 km–1 km were significantly higher than those within 0.5 km, 1 km–5 km, and within 5 km–10 km. Crime rates within 1 km–5 km were significantly higher than those within 0.5 km and within 5 km–10 km. The crime rate within 0.5 km was significantly higher than the rates within 5 km–10 km

To test Hypotheses 1a and 1b, the daily income-generating crime rates at 0.5 km, 0.5–1 km, 1–5 km, and 5–10 km radii from the pachinko parlours were compared. As the four radii had different areas, the daily income-generating crime rates were adjusted such that all areas were equal to 100 square km (Additional file 3). Table 1 shows the significant effects of these radii on the daily income-generating crime rate (*F* = 120.91, *df1* = 3, *df2* = 46,908, *p* < 0.001). Multiple comparisons showed that the daily income-generating crime rate within 0.5 km–1 km was significantly higher than the rates within 0.5 km, 1 km–5 km, and within 10 km. Crime rates within 1 km–5 km were significantly higher than those within 0.5 km and within 5 km–10 km. The crime rate within 0.5 km was significantly higher than the rates within 5 km–10 km. The results thus show that income-generating crime rates are most likely to occur within a 0.5 km–1 km radius of a pachinko parlour and that daily income-generating crime rates are less likely to occur as one moves closer to or further away from the parlour (Fig. 6).

**Table 1:** Comparison of daily income-generating crime rates according to distance from the pachinko parlour: Distance effect

	*n*	Within 0.5 km		Within 0.5 km–1 km		Within 1 km–5 km		Within 5 km–10 km	
		*M*	*S.D*	*M*	*S.D*	*M*	*S.D*	*M*	*S.D*
Newly opened then closed pachinko parlours	30	0.47	0.91	1.47	2.47	0.89	0.86	0.25	0.86
Newly opened pachinko parlours	127	0.40	0.79	1.52	6.33	0.45	0.64	0.12	0.64
Opened then closed pachinko parlours	3593	0.63	2.42	1.06	2.91	1.01	2.31	0.41	2.31
Always open pachinko parlours	3549	0.60	1.89	0.93	3.16	1.12	1.58	0.28	1.58
Always closed pachinko parlours	4430	0.87	3.42	1.47	4.06	0.66	2.70	0.83	2.70

The effects of distance on daily income-generating crime rates were significant (*F* = 120.91, *df1* = 3, *df2* = 46,908, *p* < .001). The effects of pachinko parlours on daily income-generating crime rates were also significant (*F* = 15.43, *df1* = 3, *df2* = 46,908, *p* < .001). Multiple comparisons showed that the crime rate was significantly highest in the 0.5 km–1 km range, followed by a significantly higher crime rate in the 1 km–5 km range, then a significantly higher crime rate in the 0.5 km range, and the lowest crime rate in the 5 km–10 km range; The crime rates within 0.5 km–1 km range were significantly higher than the rates within 0.5 km, 1 km–5 km, and within 5 km–10 km. Crime rates within 1–5 km were significantly higher than those within 0.5 km and within 5 km–10 km. The crime rate within 0.5 km was significantly higher than the rates within 5 km–10 km

A similar analysis was conducted for traffic crime. Daily traffic crime rates were significantly different among the distances from the pachinko parlours (*F* = 5.40, *df1* = 3, *df2* = 46,908, *p* < 0.01). Nevertheless, the results of multiple comparisons showed no significant findings except that the traffic crime rate within 1–5 km was higher than that within 0.5 km or less and 0.5–1 km. Owing to the relatively low frequency and high variance of traffic crimes, it is difficult to draw consistent conclusions about the relationship between traffic crimes and distance from pachinko parlours, although the shape of the bar graph for traffic crimes is similar to that for income-generating crimes (Additional file 6).

### Comparison of daily income-generating crime rates among pre-opening, opening, and post-closing periods of pachinko parlours

**Fig. 7:**
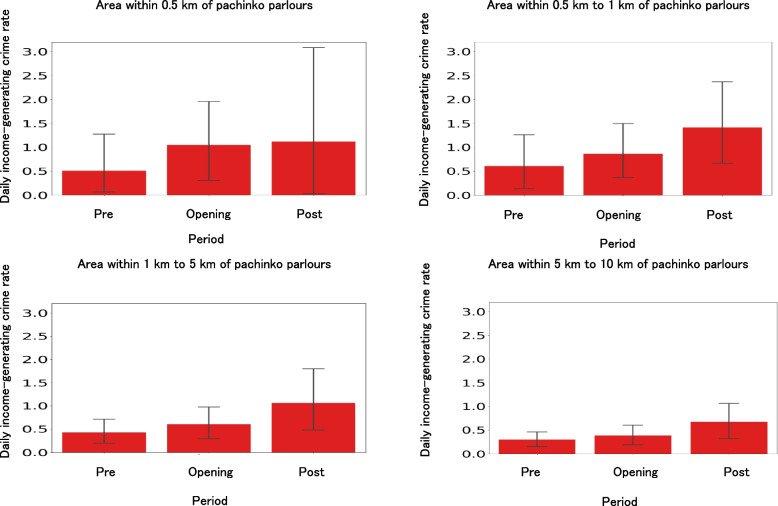
Comparison of daily income-generating crime rates before, during, and after opening of newly opened then closed pachinko parlour in Japan. Notes. Pre, opening, and post indicate the pre-opening, opening, and post-closing periods, respectively. The black bars indicate 95% confidence intervals. Daily income-generating crime rates were significantly different among the pre-opening, opening, and post-closing periods (*F* = 6.05, *df1* = 2, *df2* = 325, *p* = .002), although the rates were not significantly different among areas within 0.5 km, 0.5 km–1 km, 1 km–5 km, and 5 km–10 km distances (*F* = 1.46, *df1* = 2, *df2* = 325, *p* = .224). Multiple comparisons also showed that the daily income-generating crime rates in the opening and post-closing periods were significantly higher than those in the pre-opening period

To test Hypothesis 2a, we focused on the pre-opening, opening, and post-closing periods of 30 newly opened then closed pachinko parlours and compared the daily income-generating crime rates during each period (Fig. 7). The number of convenience stores and always open pachinko parlours near a pachinko parlour were also entered as covariates (Additional file 7). Table 2 shows the significant effects of these periods on daily income-generating crime rates (*F* = 6.05, *df1* = 2, *df2* = 325, *p* < 0.01). Figure 7 shows significantly higher income-generating crime rates during the opening and post-closing periods than the pre-opening period. Multiple comparisons also showed that the daily income-generating crime rates in the opening and post-closing periods were significantly higher than those in the pre-opening period.

**Table 2:** Comparison of daily income-generating crime rates among the pre-opening, opening, and post-closing periods of newly-opened-then-closed pachinko parlours: eriod effects

		Number of convenience stores within 5 km	Period length (in days)		
			Pre-opening	Opening	Post-closing
Total	M	229.23	615.87	889.53	838.60
	SD	402.92	537.64	569.35	570.95
By distance category		Number of always open pachinko parlours	Daily income-generating crime rate		
Within 0.5 km	M	0.33	0.51	1.05	1.84
	SD	0.55	1.72	2.23	3.61
Within 0.5–1 km	M	0.77	0.61	0.86	1.34
	SD	1.22	1.51	1.51	2.12
Within 1–5 km	M	7.93	0.43	0.60	1.00
	SD	9.29	0.72	0.98	1.68
Within 5–10 km	M	20.93	0.30	0.39	0.67
	SD	27.42	0.41	0.61	1.07

Daily income-generating crime rates were significantly different between the pre-opening, opening, and post-closing periods (*F* = 6.05, *df1* = 2, *df2* = 325, *p* = .002), although the rates were not significantly different among areas within 0.5 km, 0.5 km–1 km, 1 km–5 km, and 5 km–10 km distance (*F* = 1.46, *df1* = 2, *df2* = 325, *p* = .224)

Multiple comparisons also showed that the daily income-generating crime rates in the opening and post-closing periods were significantly higher than those in the pre-opening period

Using the period before the opening of the pachinko parlours as a baseline, the daily incidence rate of income-generating crimes during the opening period increased by 106% within 0.5 km, 41% within 0.5–1 km, 40% within 1–5 km, and 30% within 5–10 km on average (Fig. 7). Additionally, the daily incidence rate of income-generating crimes after the closing of pachinko parlours increased by 261% within 0.5 km, 120% within 0.5–1 km, 133% within 1–5 km, and 123% within 5–10 km, respectively (Fig. 7). These results indicate that income-generating crimes increased significantly during the opening and post-closing periods of the pachinko parlours, although the effects of variance need to be considered.

A similar analysis was conducted for traffic crime. Contrary to the case of income-generating crime, the daily incidence of traffic crimes was not significantly different among the three periods (Additional file 8). These results indicate that the increase in crime rates during the opening and post-closing periods is specific to income-generating crimes.

### Impact of pachinko parlour openings on the increase in local income-generating crimes

**Fig. 8:**
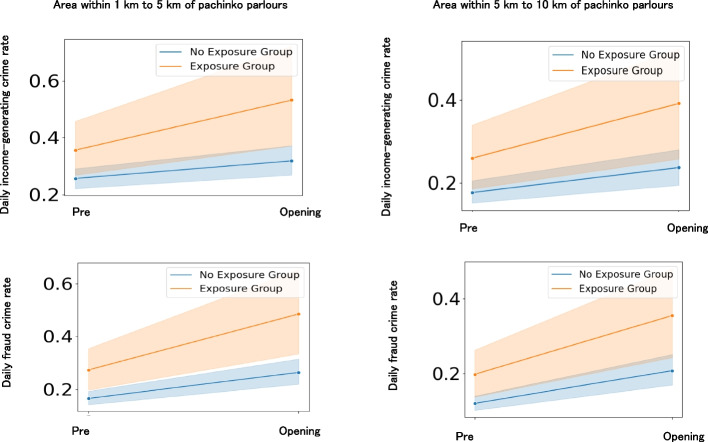
Effects of pachinko parlour openings on income-generating crimes per day. Notes. Pre and opening indicate the pre-opening and opening periods, respectively. Light colours around the lines indicate 95% confidence intervals. The exposure group refers to newly opened pachinko parlours. The no-exposure group refers to bowling alleys that never opened pachinko parlours

To examine the impact of pachinko parlour openings on the increase in local income-generating crimes (Hypothesis 2b), we compared the income-generating crime rates of newly opened pachinko parlours and bowling alleys during the pre-opening and opening periods (Fig. 8). Local residents in the newly opened pachinko parlours experienced an increase in income-generating crime rates within the 1–5 km and 5–10 km ranges during the opening period compared with the unexposed group (Table 3). Significant interaction effects on income-generating crimes were identified for small effects (effect sizes ≥ 0.2) (Table 3). Regression analysis showed that even after controlling for the number of nearby convenience stores and always open pachinko parlours, the interaction between group and time (i.e. the exposure period for the exposed group) significantly contributed to the increase in income-generating crimes (Additional file 9). A similar analysis of traffic crime did not reveal any interaction effects (Additional file 9). These results suggest that residents near pachinko parlours who are exposed to openings are more likely to encounter income-generating crimes.

**Table 3:** Effects of neighborhood exposure to pachinko parlour openings on income-generating crimes per day

Total			Exposure group		No-exposure group				
	Number of convenience stores within 5 km	M	160.18		128.79				
		SD	234.34		153.73				
			Pre-opening	Opening	Pre-opening	Opening	DD	Effect size of DD	Interaction effects
	Period Length (in days)	M	924.02	1419.98	927.28	1416.71			
		SD	675.75	675.75	684.41	684.41			
Crime type	Distance								
Income-generating crime	Within 0.5 km	M	1.03	1.25	1.29	1.13	0.38	0.19	0.71
		SD	6.56	6.83	5.21	4.11			
	Within 0.5–1 km	M	0.67	0.85	0.54	0.63	0.08	0.07	0.25
		SD	1.79	1.58	1.61	1.80			
	Within 1–5 km	M	0.36	0.53	0.26	0.32	0.11	0.27	0.19**
		SD	0.55	0.96	0.43	0.61			
	Within 5–10 km	M	0.26	0.39	0.18	0.24	0.07	0.23	0.12*
		SD	0.42	0.75	0.31	0.49			
Fraud	Within 0.5 km	M	0.95	1.27	0.74	0.77	0.29	0.19	0.52
		SD	6.84	7.08	3.07	2.93			
	Within 0.5–1 km	M	0.45	0.74	0.33	0.45	0.17	0.15	0.26
		SD	1.44	1.44	1.05	1.13			
	Within 1–5 km	M	0.27	0.48	0.17	0.26	0.11	0.27	0.18**
		SD	0.46	0.93	0.30	0.55			
	Within 5–10 km	M	0.20	0.35	0.12	0.21	0.07	0.21	0.11*
		SD	0.35	0.73	0.23	0.45			
Robbery	Within 0.5 km	M	0.03	0.02	0.11	0.08	0.03	0.12	0.04
		SD	0.07	0.09	0.51	0.30			
	Within 0.5–1 km	M	0.03	0.02	0.03	0.03	-0.01	-0.16	0.00
		SD	0.08	0.05	0.11	0.12			
	Within 1–5 km	M	0.01	0.01	0.01	0.01	0.00	0.16	0.01
		SD	0.02	0.02	0.03	0.02			
	Within 5–10 km	M	0.01	0.01	0.01	0.01	0.00	0.06	0.00
		SD	0.02	0.01	0.02	0.01			
Purse snatch	Within 0.5 km	M	0.08	0.02	0.19	0.08	0.05	0.20	0.09
		SD	0.18	0.06	0.54	0.36			
	Within 0.5–1 km	M	0.06	0.01	0.09	0.04	0.00	0.00	0.02
		SD	0.15	0.03	0.32	0.17			
	Within 1–5 km	M	0.03	0.01	0.03	0.01	0.00	0.01	0.00
		SD	0.07	0.03	0.09	0.04			
	Within 5–10 km	M	0.02	0.01	0.02	0.01	0.00	-0.05	0.00
		SD	0.05	0.02	0.06	0.02			
Theft	Within 0.5 km	M	0.15	0.11	0.59	0.44	0.11	0.10	0.16
		SD	0.38	0.36	3.01	1.89			
	Within 0.5–1 km	M	0.21	0.15	0.18	0.19	-0.07	-0.15	-0.02
		SD	0.61	0.42	0.79	1.01			
	Within 1–5 km	M	0.07	0.06	0.07	0.05	0.00	0.04	0.02
		SD	0.13	0.11	0.13	0.13			
	Within 5–10 km	M	0.05	0.04	0.04	0.03	0.00	0.06	0.00
		SD	0.09	0.08	0.07	0.05			

Differences in Differences (*DD*) implies the following: (the post-exposure mean of the exposed group – the pre-exposure mean of the exposed group) – (the post-exposure mean of the unexposed group – the pre-exposure mean of the unexposed group). Effect size of DD is equivalent to Cohen’s d_av_: 2DD / (SD_exp_diff_ + SD_unexp_diff_), where SD_exp_diff_ is the standard deviation of the differences between the post-exposure values of the exposed group and the pre-exposure values of the exposed group and SD_unexp_diff_ is the standard deviation of the differences between the post-exposure values of the unexposed group and the pre-exposure values of the unexposed group. Interaction Effect is the coefficient of ‘Treatment*Post-exposure’ in the Eq. (3) that controls for the number of convenience stores and always open pachinko parlours near a pachinko parlour

*p < .05, **: *p* < .01

Additionally, we conducted similar analyses of fraud, robbery, purse snatching, and theft among income-generating crimes. The results showed that fraud incidents were more likely to occur within the 1–5 km and 5–10 km ranges during the opening period of pachinko parlours (Table 3), whereas no significant interaction effects were found for robbery, purse snatching, and theft (Additional files 10 and 11). The increase in fraud incidents closely mirrored the increase in income-generating crimes (see Fig. 8, Table 3). Significant interaction effects on fraud were identified for small effects (effect sizes ≥ 0.2) (Table 3). These findings indicate that residents near pachinko parlours with openings are particularly vulnerable to fraud.

### Impact of pachinko parlour closings on the increase in local income-generating crimes

**Fig. 9:**
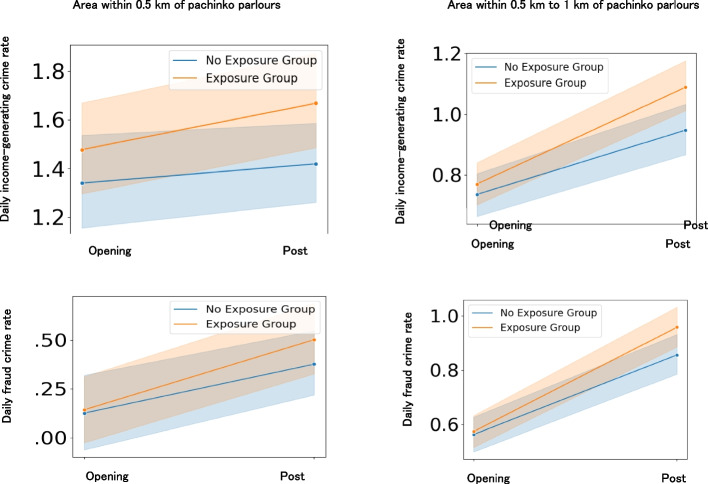
Effects of pachinko parlour closing on income-generating crimes per day. Notes. Opening and post indicate the opening and post-closing periods, respectively. Light colours around the lines indicate 95% confidence intervals. The exposure group consisted of opened and closed pachinko parlours. The no-exposure group refers to always-opened pachinko parlours that never closed

To examine the impact of pachinko parlours on the increase in local income-generating crimes (Hypothesis 2c), we compared the income-generating crime rates during the opening and post-closing periods of pachinko parlours between open-then-closed pachinko parlours and always open parlours (Fig. 2). Local residents in the open-then-closed pachinko parlours experienced an increase in income-generating crime rates within the 0.5 km, 0.5–1 km, 1–5 km, and 5–10 km ranges during the post-closing period compared to the always open group (Fig. 9), although the effect sizes of the interaction effects were lower than the small effect size guideline (0.2) (Table 4). Regression analysis showed that even after controlling for the number of nearby convenience stores and always open pachinko parlours, the interaction between group and time (i.e. the post-exposure period for the exposed group) significantly contributed to the increase in income-generating crimes (Additional file 12). A similar analysis for traffic crimes showed interaction effects within the 1–5 km range but not in other ranges (Additional file 12). These results suggest that residents near pachinko parlours who are exposed to closings are particularly vulnerable to income-generating crimes.

**Table 4:** Effects of neighborhood exposure to pachinko parlour closing on income-generating crimes per day

Total			Exposure group		No-exposure group				
	Number of convenience stores within 5 km	M	187.00		182.79				
		SD	266.02		251.84				
			Opening	Post-closing	Opening	Post-closing	DD	Effect size of DD	Interaction effects
	Period Length (in days)	M	1287.99	1056.01	1281.11	1062.52			
		SD	666.74	666.74	667.79	667.79			
Crime type	Distance								
Income-generating crime	Within 0.5 km	M	1.48	1.67	1.34	1.42	0.11	0.03	0.93**
		SD	5.33	5.39	5.33	4.54			
	Within 0.5–1 km	M	0.77	1.09	0.74	0.95	0.11	0.06	0.62**
		SD	2.08	2.41	2.13	2.42			
	Within 1–5 km	M	0.43	0.69	0.43	0.67	0.01	0.02	0.22**
		SD	0.68	1.19	0.67	1.23			
	Within 5–10 km	M	0.33	0.52	0.33	0.52	0.00	0.01	0.17**
		SD	0.50	0.89	0.50	0.89			
Fraud	Within 0.5 km	M	1.14	1.50	1.12	1.38	0.11	0.03	0.83**
		SD	4.63	4.84	5.14	4.72			
	Within 0.5–1 km	M	0.57	0.96	0.56	0.86	0.09	0.06	0.55**
		SD	1.73	2.15	1.92	2.09			
	Within 1–5 km	M	0.33	0.62	0.33	0.60	0.02	0.03	0.21**
		SD	0.59	1.15	0.59	1.13			
	Within 5–10 km	M	0.26	0.48	0.26	0.47	0.01	0.02	0.17**
		SD	0.44	0.86	0.44	0.85			
Robbery	Within 0.5 km	M	0.08	0.07	0.06	0.06	-0.02	-0.07	0.01
		SD	0.35	0.31	0.23	0.30			
	Within 0.5–1 km	M	0.03	0.03	0.04	0.03	0.01	0.03	0.02*
		SD	0.14	0.15	0.34	0.15			
	Within 1–5 km	M	0.02	0.02	0.02	0.01	0.00	0.05	0.00**
		SD	0.03	0.03	0.03	0.03			
	Within 5–10 km	M	0.01	0.01	0.01	0.01	0.00	0.02	0.00**
		SD	0.02	0.01	0.02	0.01			
Purse snatch	Within 0.5 km	M	0.18	0.05	0.18	0.06	0.00	-0.01	0.03
		SD	0.50	0.18	0.56	0.24			
	Within 0.5–1 km	M	0.09	0.03	0.10	0.04	0.00	0.01	0.03*
		SD	0.26	0.11	0.47	0.17			
	Within 1–5 km	M	0.04	0.02	0.04	0.02	0.00	0.00	0.00
		SD	0.11	0.04	0.11	0.04			
	Within 5–10 km	M	0.03	0.01	0.03	0.01	0.00	0.03	0.00**
		SD	0.06	0.02	0.07	0.02			
Theft	Within 0.5 km	M	0.37	0.29	0.26	0.19	-0.02	-0.02	0.02
		SD	1.87	1.63	1.49	1.24			
	Within 0.5–1 km	M	0.17	0.15	0.15	0.10	0.02	0.05	0.05*
		SD	0.66	0.75	0.61	0.42			
	Within 1–5 km	M	0.07	0.06	0.07	0.06	0.00	0.05	0.02**
		SD	0.13	0.14	0.12	0.12			
	Within 5–10 km	M	0.05	0.04	0.05	0.04	0.00	0.01	0.00
		SD	0.08	0.06	0.08	0.06			

Differences in Differences (*DD*) implies the following: (the post-exposure mean of the exposed group – the pre-exposure mean of the exposed group) – (the post-exposure mean of the unexposed group – the pre-exposure mean of the unexposed group). Effect size of DD is equivalent to Cohen’s d_av_: 2DD / (SD_exp_diff_ + SD_unexp_diff_), where SD_exp_diff_ is the standard deviation of the differences between the post-exposure values of the exposed group and the pre-exposure values of the exposed group and SD_unexp_diff_ is the standard deviation of the differences between the post-exposure values of the unexposed group and the pre-exposure values of the unexposed group. Interaction Effect is the coefficient of ‘Treatment*Post-exposure’ in the Eq. (3) that controls for the number of convenience stores and always open pachinko parlours near a pachinko parlour

*p < .05, **p <.01

Additionally, we conducted similar analyses of fraud, robbery, snatching, and theft among income-generating crimes. The results showed that fraud incidents were more likely to occur within the 0.5 km, 0.5–1 km, 1–5 km, and 5–10 km ranges during the post-closing period of pachinko parlours, although the effect sizes of the interaction effects were lower than the small effect size guideline (0.2) (Table 4). Robbery, snatching, and theft were also more likely to increase in some of these ranges during the post-closing period (Additional files 13 and 14). The increase in fraud incidents closely mirrored the increase in income-generating crimes (see Fig. 9). These findings indicate that residents near pachinko parlours exposed to closures are particularly vulnerable to fraud.

### Trends in income-generating crime rates over time

To test Hypothesis 2d, we examined trends over time in income-generating crime rates by pachinko parlour class. We focused on three classes of pachinko parlours—always open, always closed, and opened-then-closed—because the 95% confidence intervals of the income-generating crime rates among these classes were stable. We controlled for the years when income-generating crimes occurred. Furthermore, the number of convenience stores and always open pachinko parlours near a pachinko parlour were controlled for (Additional file 15). Table 5 shows the significant effects of these classes on the daily income-generating crime rates.

**Table 5:** Comparison of 6.5-year income-generating crime rates by pachinko parlour types

Distance	Class		Number of convenience stores within 5 km	Number of always open pachinko parlours in the neighborhood	Daily income-generating crime rate						
					2017 (365 days)	2018 (365 days)	2019 (365 days)	2020 (366 days)	2021 (365 days)	2022 (365 days)	2023 (153 days)
Within 0.5 km	always closed (*n* = 4430)	M	213.10	0.58	1.91	1.54	1.75	1.68	1.98	1.95	1.81
		S.D	316.40	1.20	7.66	6.26	6.73	5.87	6.10	6.16	5.97
	opened-then closed (*n* = 3593)	M	187	0.45	1.21	1.13	1.25	1.21	1.49	1.52	1.41
		S.D	266.02	1.02	5.24	5.01	5.23	4.83	5.36	5.31	4.38
	always open (*n* = 3549)	M	182.8	1.44	1.23	1.1	1.17	1.18	1.35	1.34	1.19
		S.D	251.84	1.04	5.86	5.25	5.38	5.44	5.45	4.63	3.51
Within 0.5–1 km	always closed (*n* = 4430)	M	213.1	0.47	0.89	0.75	0.85	0.95	1.2	1.22	1.19
		S.D	316.4	0.97	2.64	2.34	2.51	2.38	2.74	2.75	2.91
	opened-then closed (*n* = 3593)	M	187	0.39	0.7	0.63	0.71	0.82	1.04	1.1	1.08
		S.D	266.02	0.81	2.27	2.04	2.23	2.25	2.53	2.57	2.7
	always open (*n* = 3549)	M	182.8	0.39	0.69	0.62	0.7	0.7	0.91	0.95	0.94
		S.D	251.84	0.8	2.32	2.2	2.29	1.98	2.33	2.24	2.33
Within 1–5 km	always closed (*n* = 4430)	M	213.1	9.9	0.4	0.38	0.47	0.57	0.8	0.84	0.76
		S.D	316.4	12.38	0.59	0.63	0.81	1	1.51	1.53	1.3
	opened-then closed (*n* = 3593)	M	187	9.35	0.38	0.37	0.43	0.5	0.7	0.76	0.73
		S.D	266.02	11.56	0.55	0.59	0.73	0.88	1.34	1.38	1.23
	always open (*n* = 3549)	M	182.8	9.34	0.37	0.37	0.43	0.48	0.67	0.72	0.7
		S.D	251.84	11.84	0.53	0.6	0.75	0.83	1.27	1.31	1.21
Within 5–10 km	always closed (*n* = 4430)	M	213.1	22.11	0.3	0.29	0.33	0.42	0.59	0.63	0.57
		S.D	316.4	29.39	0.43	0.43	0.53	0.73	1.08	1.13	0.96
	opened-then closed (*n* = 3593)	M	187	21.24	0.29	0.28	0.32	0.39	0.53	0.58	0.55
		S.D	266.02	28	0.41	0.41	0.5	0.67	0.97	1.02	0.91
	always open (*n* = 3549)	M	182.8	21.78	0.29	0.29	0.32	0.38	0.52	0.57	0.55
		S.D	251.84	28.8	0.41	0.43	0.51	0.63	0.92	0.98	0.92

*n* = 11,572

Income-generating crime rates differed significantly among the pachinko parlour classes (*F* = 167.14, *df1* = 2, *df2* = 324,002,* p* < .001). Significant between-group differences were also found in the distances from pachinko parlours (*F* = 1161.63, *df1* = 3, *df2* = 324,002,* p* < .001) and years (*F* = 113.92, *df1* = 6, *df2* = 324,002, *p* < .001)

Multiple comparisons also showed that the income-generating crime rates of closed pachinko parlours were significantly higher than those of open and always-opened pachinko parlours. Furthermore, the number of open-then closed pachinko parlours was significantly higher than that of always open pachinko parlours

**Fig. 10:**
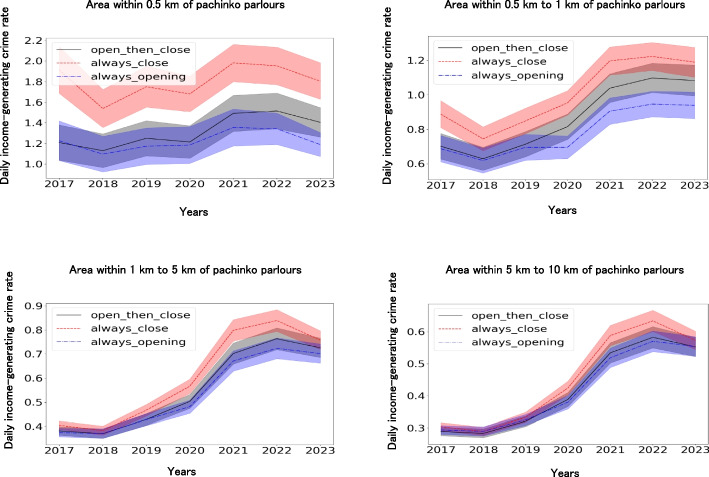
Comparison of daily income-generating crime rates from 2017 to 2023 among pachinko parlours in Japan. Notes. Lightly painted areas indicate 95% confidence intervals. Income-generating crime rates differed significantly among the pachinko parlour classes (*F* = 167.14, *df1* = 2, *df2* = 324,002, *p* < .001). Significant between-group differences were also found in the distances from pachinko parlours (*F* = 1161.63, *df1* = 3, *df2* = 324,002, *p* < .001) and years (*F* = 113.92, *df1* = 6, *df2* = 324,002, *p* < .001). Multiple comparisons also showed that the income-generating crime rates of closed pachinko parlours were significantly higher than those of opened and always open pachinko parlours. Furthermore, the number of opened then closed pachinko parlours was significantly higher than that of always open pachinko parlours

Figure 10 shows a significantly higher income-generating crime rate in always closed pachinko parlours than in always open pachinko parlours. Figure 10 also shows significantly higher income-generating crime rates in open-then-closed pachinko parlours than in always open parlours. A closer look at the daily income-generating crime rates for the 0.5 km, 0.5–1 km, and 1–5 km radii of pachinko parlours in Fig. 10 showed that even though opened-then-closed pachinko parlours’ income-generating crime rate in 2017 approximated that of always-opened pachinko parlours, the income-generating crime rate for opened-then-closed pachinko parlours increased as the year progressed, and this rate in 2023 approached that of the always closed pachinko parlours.

A similar analysis was conducted for traffic crime. The results for traffic crimes were similar to those for income-generating crimes (Additional file 16). However, it was difficult to identify consistent trends because there were no consistent increases associated with specific classes of pachinko parlours (Additional file 17). This suggests that closing pachinko parlours increases the incidence of income-generating crimes rather than traffic crimes.

## Discussion

### Primary findings

This study examined the proximity effects of pachinko parlours on daily income-generating crime rates by considering individual nationwide crime records spanning 6.5 years in Japan. As the granularity of the present data involved individual crime cases, longitude/latitude, and date units, they contained rich information on the association between crime rates and gambling venues [6, 8]. Furthermore, nationwide datasets of pachinko parlours in Japan are expected to enrich EGM research data [11, 37, 38].

This study demonstrated that pachinko parlours in Japan, unlike those in Western countries [5, 64], are uniformly distributed across the nation and not concentrated in impoverished areas. It was also observed that income-generating crime rates are higher in wealthy areas [41]. Therefore, opening pachinko parlours in wealthy regions can be considered a risk factor for increased income-generating crime rates. This is because wealthy areas tend to have more valuable items that can be stolen [65], and pachinko parlours can induce gambling disorders among the local residents [9, 17], some of whom may commit income-generating crimes as a consequence of gambling-related harm [20–24].

Consistent with the regional exposure theory [13, 15], opening pachinko parlours increased income-generating crimes. Consistent with Hypothesis 1a, areas within 1–5 km of a pachinko parlour were more prone to income-generating crime than areas within 5–10 km. Additionally, supporting Hypothesis 2a, income-generating crimes were more likely to occur during the opening of pachinko parlours than during the pre-opening period. In line with Hypothesis 2b, areas exposed to the opening of a pachinko parlour experienced an increase in income-generating crimes during the opening period compared with unexposed areas. These results can be interpreted as follows: residents living near pachinko parlours are highly likely to engage in gambling activities [16], which increases the risk of gambling disorder [9, 17] and makes them highly likely to commit income-generating crimes such as gambling-related harm [20–24, 28, 29].

Consistent with the rational choice perspective of income-generating perpetrators [14], the closing of pachinko parlours increased income-generating crimes. Supporting Hypothesis 2c, areas exposed to the closing of a pachinko parlour experienced an increase in income-generating crimes during the post-closing period compared with unexposed areas. Furthermore, consistent with Hypothesis 2d, there was a trend towards a gradual yearly increase in income-generating crimes following the closure of a pachinko parlour. Moreover, consistent with hypothesis 1b, the area within 0.5–1 km of a pachinko parlour was more prone to income-generating crimes than the area within 0.5 km. These results align with the understanding that income-generating perpetrators may avoid committing crimes too close to frequently used facilities to reduce the risk of their identity being revealed [25] and instead commit crimes slightly further away [26] or at facilities they used in the past but no longer frequent [27].

We speculate that the increase in income-generating crimes following the closure of pachinko parlours compared to when they are open can be explained by factors that maintain income-generating crimes due to gambling disorder [66] and factors that reduce the risk of arrest [14]. First, the opening of pachinko parlours facilitates frequent visits and extended playing time among local residents [16]; thereby, increasing the likelihood of developing gambling disorders [9, 17]. Some of those who develop gambling disorders commit income-generating crimes to obtain money for gambling [22, 23]. Thus, even after the pachinko parlour suddenly closes down, residents who are already addicted to gambling and have committed income-generating crimes will not stop gambling [18]. Therefore, although they do not visit the closed-down pachinko parlour, they will continue to frequently visit other nearby pachinko parlours that are still open [19]. As they continue gambling, they tend to seek more money for it [21]. Since they are familiar with the local area, they find it easier to commit income-generating crimes [25–27]. Thus, a certain number of residents who are addicted to gambling and commit income-generating crimes will remain in the vicinity of the closed-down pachinko parlour [9, 17], thus maintaining the level of income-generating crimes to comparable to those observed during the opening of the pachinko parlour.

Additionally, although residents who are addicted to gambling and commit income-generating crimes find it easier to commit crimes in the areas they are familiar with [26], they are less likely to commit income-generating crimes near their own home or their current frequently visited pachinko parlour due to their fear of exposing their identity [25]. However, if the pachinko parlour is one they used to frequently visit, they may be familiar with the area; furthermore, fewer people will know them since they no longer go there, thus reducing the risk of identity exposure [27]. In short, income-generating criminals with gambling disorders consider the area around a pachinko parlour they used to frequently visit but no longer do as an optimal environment for committing income-generating crimes, as it allows them to set up escape routes easily and minimizes the risk of exposure [14]. Consequently, the decreased likelihood of arrest in these environments contributes to an increase in income-generating crimes in the vicinity of closed pachinko parlours.

Hypotheses 2b and 2c also indicated that fraud was particularly prevalent near pachinko parlours. In Japan, 72.2% of fraud perpetrators are under the age of 30 [55]. Because of their youth, they are more likely to take financial risks [67] and are vulnerable to gambling disorders [68]. Additionally, organised crime groups in Japan (yakuza) are involved in directing fraud perpetrators [55]. They use social networking sites to offer illegal, high-paying, part-time jobs to young people in financial distress [69]. Furthermore, 85.7% of the fraud victims are older adults, aged 65 or older [55]. Based on these findings, it can be inferred that the opening of pachinko parlours leads to a situation in which young residents in the vicinity become addicted to gambling. Through social network services, the yakuza introduce illegal high-paying jobs to young people who are short on money, resulting in them becoming fraud perpetrators targeting older residents in the neighbourhood. Thus, pachinko parlours’ operations may contribute to an increase in gambling disorders [16, 28, 29], fraud perpetration among young people, and fraud victims among older adults in the vicinity, spreading public harm.

To prevent income-generating crimes, especially fraud, near pachinko parlours, it is crucial to avoid building them in residential areas. Particularly, when establishing new pachinko parlours, it would be advisable to obtain approval not only from operating companies and the police but also from residents living within 0.5 to 5 km of the proposed location. For currently operating pachinko parlours, it is desirable to monitor local income-generating crimes, especially fraud, and for operating companies to compensate for any damage.

The findings of this study may also be applied to other popular gambling facilities in Japan, such as horse and bicycle racing tracks [31]. Therefore, it is necessary to monitor whether income-generating crimes are increasing near these gambling facilities and to seek compensation from operators for any criminal damage experienced by local residents. Additionally, the amount and time spent on online gambling may be related to income-generating crimes rather than the location [70]. Hence, it is desirable to monitor spending and usage time and set appropriate limits for excessive amounts or durations.

### Limitations

Although this study considered individual longitudinal crime case records spanning 6.5 years, the dataset had four deficiencies. First, the crime data used in this study represent only a small portion. For example, in terms of theft, the study handled 51,687 cases over 6.5 years from the GACCOM dataset [53], whereas the official record is 381,769 cases per year [71], which means that this study covers only approximately 1/48th of the total theft. Therefore, to ensure the representativeness of crime data, it will be necessary to reanalyse the official crime data in future studies.

Second, the incidence rates of crimes per day for the pre-opening periods were mostly limited because many pachinko parlours have closed in recent years and new ones are rare [12, 34, 36]. However, using the DD approach [60, 61], this study was able to estimate the increase in crime rates in areas exposed to the opening of pachinko parlours.

Third, this study did not include pedestrian flow data. Therefore, we could not examine the increase in crime rates according to population density. The effect of population density was partially controlled by the number of nearby convenience stores; however, future analyses should include pedestrian flow data.

Fourth, this study assumed that many fraud cases involved physical proximity between the victim and perpetrator, but they also included some cases in which the victim and perpetrator were physically distant [71]. In the future, it will be necessary to analyse only fraud that involves physical proximity.

## Conclusions

The current study examined the proximity effects of pachinko parlours on daily income-generating crime rate spanning 6.5 years and clarified the following conclusions: First, the opening of gambling venues increased income-generating crime in the opening period more than in the pre-opening period, even after controlling for the number of convenience stores and always open pachinko parlours. This increase may be attributed to the physical proximity between gambling venues and residential neighbourhoods [6, 9, 22]. Second, even after the closing of gambling venues, income-generating crimes increased. This suggests that physical proximity to gambling venues is associated with a higher incidence of persistent long-term gambling disorders. Furthermore, potential income-generating perpetrators with gambling disorders know the area near closed pachinko parlours and do not visit them, so they can easily prepare various escape routes and have a low risk of being unmasked. These environmental conditions may make them commit income-generating crimes more easily [25–27]. Third, it is desirable to gain an understanding of local residents in advance when opening new gambling establishments, and it would be better to proceed with the assumption that the company will be responsible for a certain amount of compensation for criminal damage to local residents. Finally, close monitoring of how much income-generating crime increases with the opening or closing of pachinko parlours is required to take appropriate preventive and response measures near the parlours [1, 7], especially for income-generating crimes. These steps are important for opening or closing gambling venues.

### Supplementary Information

Supplementary Material 1.

Supplementary Material 2.

Supplementary Material 3.

Supplementary Material 4.

Supplementary Material 5.

Supplementary Material 6.

Supplementary Material 7.

Supplementary Material 8.

Supplementary Material 9.

Supplementary Material 1.

Supplementary Material 11.

Supplementary Material 12.

Supplementary Material 13.

Supplementary Material 14.

Supplementary Material 15.

Supplementary Material 16.

Supplementary Material 17.

## Data Availability

The data used in this study are available online (1–4) (http://koko-pachi.com/hall/data.0.1.htm, https://www.gaccom.jp/, https://www.reinfolib.mlit.go.jp/, https://www.navitime.co.jp/). The website tracking the opening and closing of pachinko parlours (http://koko-pachi.com/hall/data.0.1.htm) is currently temporarily closed due to increased maintenance costs. The site's owner is considering launching a new version under a different domain name to reduce these expenses. Researchers needing access to data from the closed site should reach out directly to the owner through their X (formerly Twitter) account (@kokopachi). Website with information on crimes in Japan (https://www.gaccom.jp/) and website with information on land in Japan (https://www.gaccom.jp/) can be accessed from within Japan. Although these websites are basically inaccessible from overseas, researchers can access them from abroad by using a virtual private network and going through a server in Japan. The datasets used and/or analysed in the current study are available from the corresponding author upon reasonable request.

## Abbreviations

ANCOVA: Analysis Of Covariance
ANOVA: Analysis Of Variance
API: Application Programming Interface
DD: Differences-in-Differences analysis
EGM: Electronic Gambling Machine
SDK: Software Development Kit
JS: Jensen-Shannon
