# Supplementary material for: Effect of pachinko parlour openings and closings on neighbourhood income-generating crimes in Japan: 6.5 years of observations
Source: BMC Public Health. 2024 Jul 16;24:1905. doi: 10.1186/s12889-024-19373-1 (PMC11250958; doi:10.1186/s12889-024-19373-1)
Supplement: Supplementary file 1 — Supplementary Material 1. [file 12889_2024_19373_MOESM1_ESM.docx]

Additional file 1. Extracting the type, date, and location of a crime from publicly available criminal act information.

Information on a purse-snatching incident (3-chome, Daikoku-cho, Toyonaka City)

On May 12, around 2:25 p.m., a man on a bicycle stole a bag containing groceries from a passerby’s back basket on a street near 3-chome, Daikoku-cho, Toyonaka City, and fled the scene.

Extract Date of Occurrence

12-MAY-YYYY

: The offender is a thin man wearing a grass-patterned white shirt and hat.

The year of the crime is missing and will be supplemented from the reporting date.

12-May-2023

Please be careful not to become a victim of such crimes when wearing anti-snatch-and-grab covers.

Date and time of report 12 May 2023 23:21

Crime type

Identify the crime as an income-generating crime from the crime type.

purse snatching

theft

Identify the crime location.

The location was converted into its corresponding longitude and latitude.

135.469638, 34.739347

Area of occurrence

3 Daikoku-cho, Toyonaka City, Osaka

*Notes*. The original text was in Japanese; it was translated into English.
