# Supplementary material for: Effect of pachinko parlour openings and closings on neighbourhood income-generating crimes in Japan: 6.5 years of observations
Source: BMC Public Health. 2024 Jul 16;24:1905. doi: 10.1186/s12889-024-19373-1 (PMC11250958; doi:10.1186/s12889-024-19373-1)
Supplement: Supplementary file 2 — Supplementary Material 2. [file 12889_2024_19373_MOESM2_ESM.docx]

Additional file 2. Comparison of the number of crimes published in Gaccom by year

| Year | Frequency |
| --- | --- |
| 1999 | 3 |
| 2013 | 11 |
| 2014 | 37 |
| 2015 | 92 |
| 2016 | 37,253 |
| 2017 | 98,345 |
| 2018 | 113,784 |
| 2019 | 117,253 |
| 2020 | 125.310 |
| 2021 | 140,708 |
| 2022 | 148,345 |
| 2023 | 50,175^a^ |

*Notes*. ^a^: The sampling period of 2023 is from 1/JAN/2023 to 3/JUN/2023; hence, the amount of data in 2023 is less than that in other years. The reason for this sampling period was that Gaccom started its information service related to public safety in December 2016, and there was a significant lack of data prior to this date. Since the service started in January 2017, the data from that point onwards are much more comprehensive; therefore, this study used data from 1 January 2017 to 3 June 2023.
