# Supplementary material for: Effect of pachinko parlour openings and closings on neighbourhood income-generating crimes in Japan: 6.5 years of observations
Source: BMC Public Health. 2024 Jul 16;24:1905. doi: 10.1186/s12889-024-19373-1 (PMC11250958; doi:10.1186/s12889-024-19373-1)
Supplement: Supplementary file 3 — Supplementary Material 3. [file 12889_2024_19373_MOESM3_ESM.docx]

Additional file 3. Parameter that adjusts the crime rate in the neighbourhood of a pachinko parlour based on square footage.

Area within 0.5 km from pachinko parlour: 0.79.

Parameter to adjust the above area to an area of 100 km sq: 127.32.

Area within 0.5 km to 1 km from pachinko parlour: 2.36

Parameter to adjust the above area to an area of 100 km sq: 42.44.

5-10

km

1-5

km

0.5-1

km

0.5

km

Area within 1 km to 5 km from pachinko parlour: 75.40

Parameter to adjust the above area to an area of 100 km sq: 1.33.

Area within 5 km to 10 km from pachinko parlour: 235.62

Parameter to adjust the above area to an area of 100 km sq: 0.42.

A pachinko parlour
