# Supplementary material for: Effect of pachinko parlour openings and closings on neighbourhood income-generating crimes in Japan: 6.5 years of observations
Source: BMC Public Health. 2024 Jul 16;24:1905. doi: 10.1186/s12889-024-19373-1 (PMC11250958; doi:10.1186/s12889-024-19373-1)
Supplement: Supplementary file 4 — Supplementary Material 4. [file 12889_2024_19373_MOESM4_ESM.docx]

Additional file 4. Correlation between land prices and number of convenience stores in Japan

| Areas within 0.5 km radius of lands (r = 0.73) | Areas within 1 km radius of lands (r = 0.82) |
| --- | --- |
| 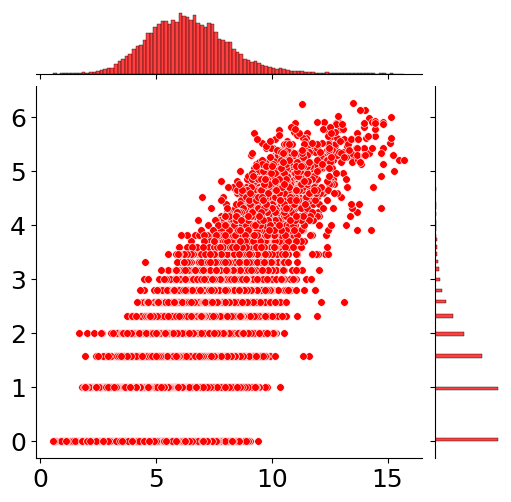  Land price  (log2)  Number of convenience stores  (log2) | 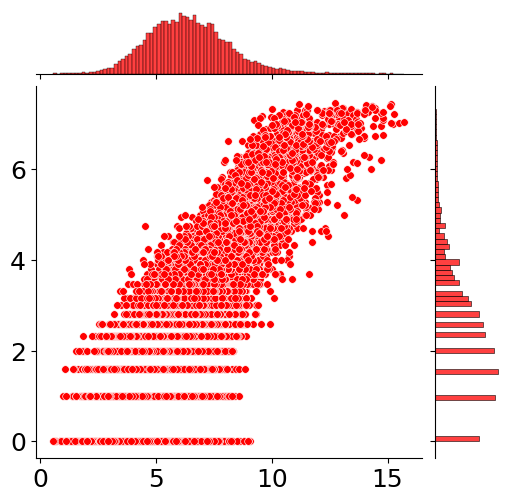  Land price  (log2)  Number of convenience stores  (log2) |
| Areas within 5 km radius of lands (r = 0.83) | Areas within 10 km radius of lands (r = 0.80) |
| 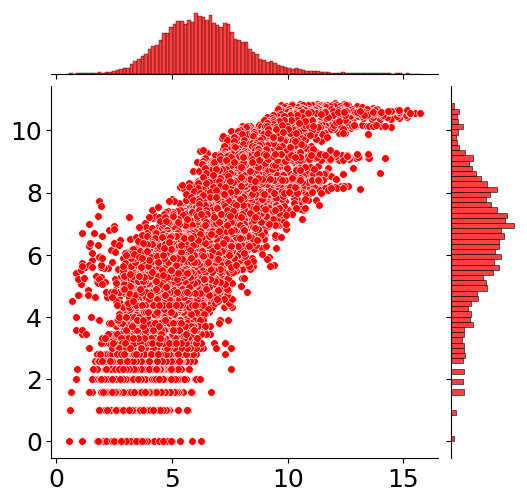  Land price  (log2)  Number of convenience stores  (log2) | 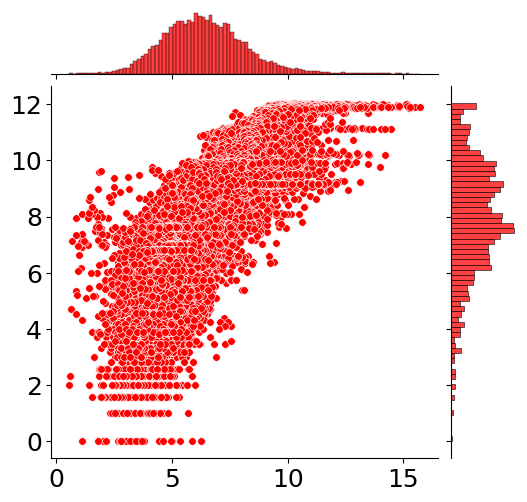  Land price  (log2)  Number of convenience stores  (log2) |

n = 25,993

*Notes.* Land value is the price per square meter, log-transformed using base 2. For example, a land price of 15 was obtained by multiplying 2 with the 15th power by 100 yen (3,276,800 Japanese yen). At JPY 157 per Euro, this would be 20,871 Euros. If one were to purchase a 100 square meter plot of land in that location, enough to build a small house, the price of the land would be 2,087,133 Euros. The number of convenience stores was also log-transformed to a base number of two after adding one. If the number of convenience stores within a 5 km radius is 10, it indicates that there are approximately 1024 convenience stores, that is, to the 10th power of 2.
