# Supplementary material for: Effect of pachinko parlour openings and closings on neighbourhood income-generating crimes in Japan: 6.5 years of observations
Source: BMC Public Health. 2024 Jul 16;24:1905. doi: 10.1186/s12889-024-19373-1 (PMC11250958; doi:10.1186/s12889-024-19373-1)
Supplement: Supplementary file 5 — Supplementary Material 5. [file 12889_2024_19373_MOESM5_ESM.docx]

Additional file 5. Correlation coefficients of daily income-generating crime with the number of convenience stores and opening pachinko parlours near pachinko parlours

|  | The number of convenience stores near the pachinko parlour (log2) | The number of always open pachinko parlours near the pachinko parlour (log2) |
| --- | --- | --- |
| Daily income-generating crime rate in newly-opened-then closed pachinko parlours | 0.36 | 0.14 |
| Daily income-generating crime rate in newly opened pachinko parlours | 0.24 | 0.03 |
| Daily income-generating crime rate in open-then closed pachinko parlours | 0.30 | 0.22 |
| Daily income-generating crime rate in always open pachinko parlours | 0.31 | 0.25 |
| Daily income-generating crime rate in always closed pachinko parlours | 0.31 | 0.24 |
