# Supplementary material for: Effect of pachinko parlour openings and closings on neighbourhood income-generating crimes in Japan: 6.5 years of observations
Source: BMC Public Health. 2024 Jul 16;24:1905. doi: 10.1186/s12889-024-19373-1 (PMC11250958; doi:10.1186/s12889-024-19373-1)
Supplement: Supplementary file 6 — Supplementary Material 6. [file 12889_2024_19373_MOESM6_ESM.docx]

Additional file 6. Comparison of daily traffic crime rates within 0.5 km, 0.5, 1, 1, 5, and 5 km to 10 km of pachinko parlours in Japan

| Newly opened then closed pachinko parlour (n = 30) | Newly opened pachinko parlour (n = 127) | Open then closed pachinko parlour (n = 3593) |
| --- | --- | --- |
| 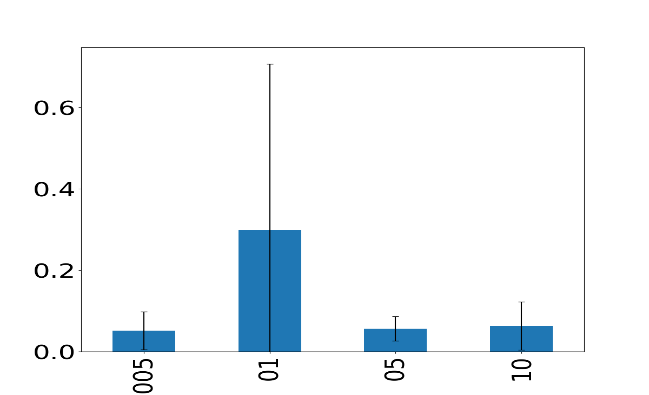  Distance from pachinko parlourparlour  Daily traffic crime rate | 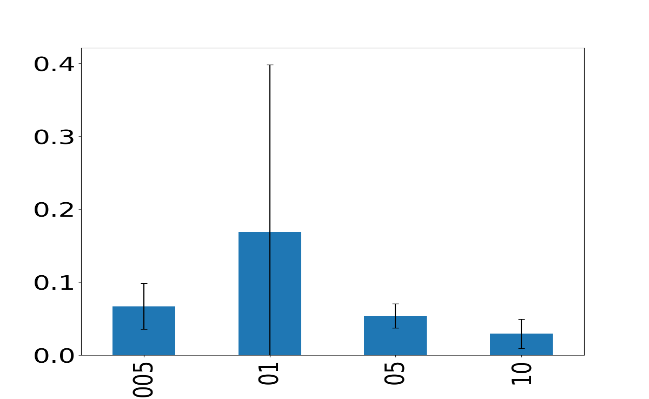  Distance from pachinko parlour  Daily traffic crime rate | 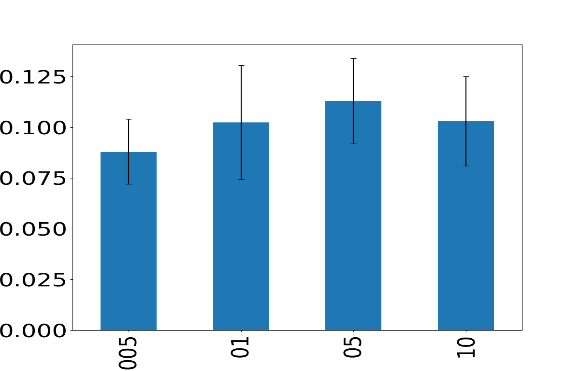  Daily traffic crime rate  Distance from pachinko parlour |
| Always open pachinko parlour (n = 3549) | Always closed pachinko parlour (n = 4430) |  |
| 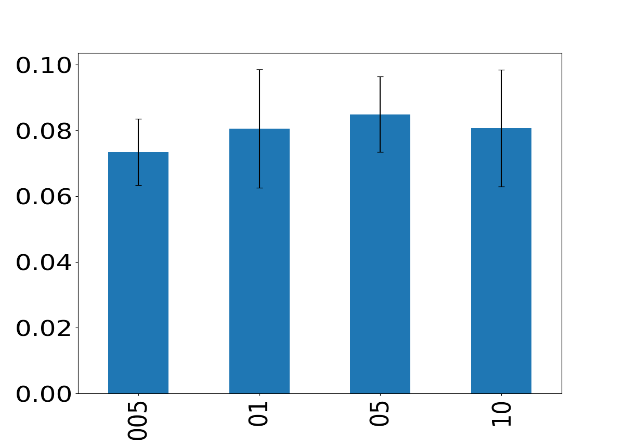  Distance from pachinko parlour  Daily traffic crime rate | 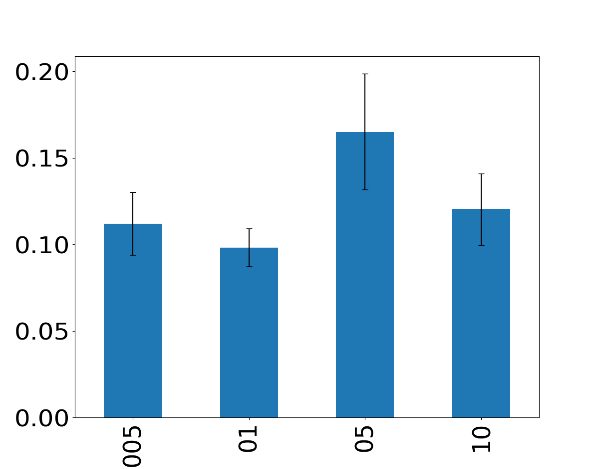  Distance from pachinko parlour  Daily traffic crime rate |  |

The effects of distance from pachinko parlours on daily traffic crime rates were significant (*F*=5.40, *df1*=3, *df2*=46908, *p* < .001). The distance effects from pachinko parlours on daily traffic crime rates were also significant (*F*=5.40, *df1*=3, *df2*=46908, *p* < .01). However, multiple comparisons yielded mixed results. Although the crime rates within 1 km-5 km were significantly higher than the rates within 0.5 km and within 0.5–1 km, no significant results were obtained for the other relationships.
