# Supplementary material for: Effect of pachinko parlour openings and closings on neighbourhood income-generating crimes in Japan: 6.5 years of observations
Source: BMC Public Health. 2024 Jul 16;24:1905. doi: 10.1186/s12889-024-19373-1 (PMC11250958; doi:10.1186/s12889-024-19373-1)
Supplement: Supplementary file 7 — Supplementary Material 7. [file 12889_2024_19373_MOESM7_ESM.docx]

Additional file 7. Additional values of Table 2

|  |  | Number of convenience stores within 5 km | Period length (in days) | | |
| --- | --- | --- | --- | --- | --- |
|  |  |  | Pre-opening | Opening | Post-closing |
| Total | Min | 1.00 | 46.00 | 6.00 | 19.00 |
|  | Max | 1589.00 | 2145.00 | 2132.00 | 2083.00 |
| By distance category |  | Number of always open pachinko parlours | Daily income-generating crime rate | | |
| Within 0.5 km | Min | 0.00 | 0.00 | 0.00 | 0.00 |
|  | Max | 2.00 | 8.38 | 9.82 | 15.40 |
| Within 0.5–1 km | Min | 0.00 | 0.00 | 0.00 | 0.00 |
|  | Max | 4.00 | 6.90 | 5.89 | 8.87 |
| Within 1–5 km | Min | 0.00 | 0.00 | 0.00 | 0.00 |
|  | Max | 36.00 | 2.91 | 3.65 | 6.54 |
| Within 5–10 km | Min | 0.00 | 0.00 | 0.00 | 0.00 |
|  | Max | 95.00 | 1.29 | 2.21 | 3.68 |

*Notes.* The daily income-generating crime rates in the neighborhood of the pachinko parlors were significantly associated with the number of convenience stores in the neighborhood of the pachinko parlors(*F*=12.85, *df1*=1, *df2*=325, *p* < .001), but not the number of always open pachinko parlors open (*F*=0.81, *df1*=1, *df2*=325, *p* = .35).
