# Supplementary material for: Effect of pachinko parlour openings and closings on neighbourhood income-generating crimes in Japan: 6.5 years of observations
Source: BMC Public Health. 2024 Jul 16;24:1905. doi: 10.1186/s12889-024-19373-1 (PMC11250958; doi:10.1186/s12889-024-19373-1)
Supplement: Supplementary file 8 — Supplementary Material 8. [file 12889_2024_19373_MOESM8_ESM.docx]

Additional file 8. Comparison of daily traffic crime rates before, during, and after opening newly opened and closed pachinko parlours in Japan.

| Area within 0.5 km of pachinko parlours | Area within 0.5 km to 1 km of pachinko parlours |
| --- | --- |
| 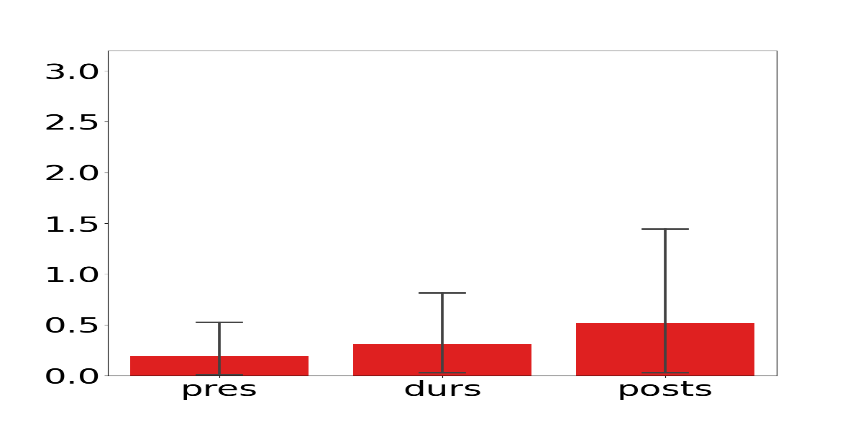  Pre Opening Post  Period  Daily traffic crime rate | 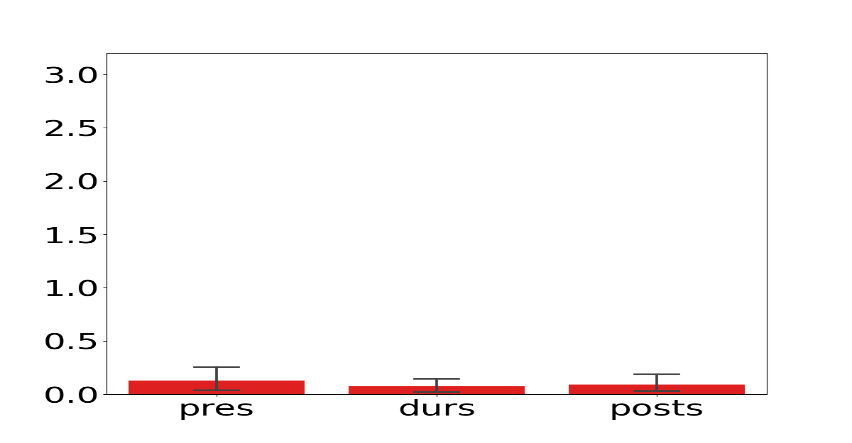  Pre Opening Post  Period  Daily traffic crime rate |
| Area within 1 km to 5 km of pachinko parlours | Area within 5 km to 10 km of pachinko parlours |
| 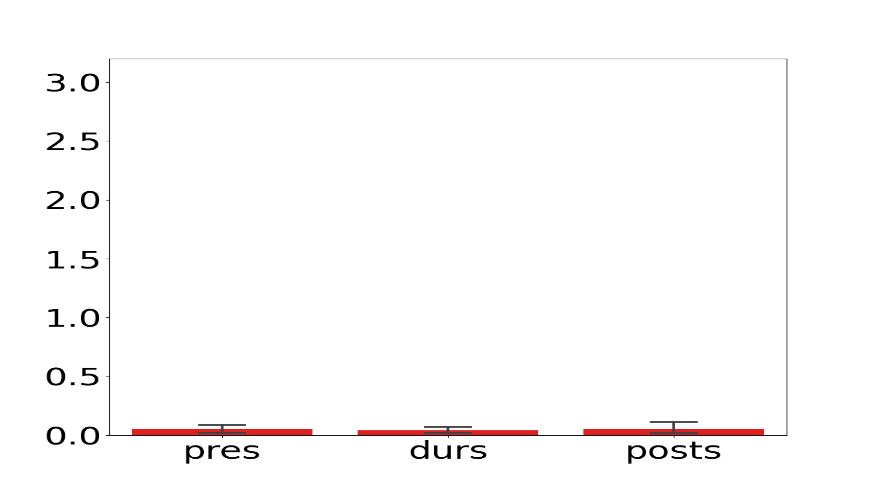  Period  Pre Opening Post  Daily traffic crime rate | 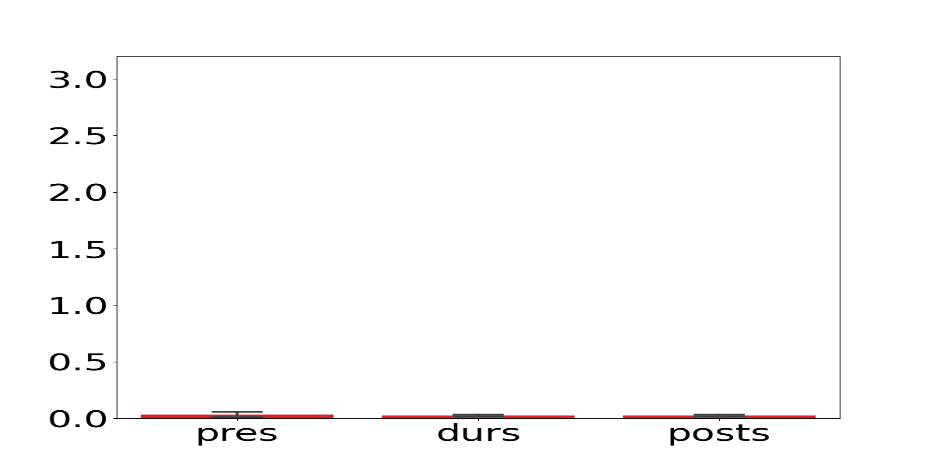  Daily traffic crime rate  Pre Opening Post  Period |

n = 30 pachinko parlours × 3 periods

*Notes.* Pre, opening, and post indicate the pre-opening, opening, and post-closing periods, respectively. The black bars indicate 95% confidence intervals. Daily traffic crime rates were not significantly different among the pre-opening, opening, and post-closing periods (*F*=0.01, *df1*=2, *df2*=325, *p* =.984), although they were significantly different among areas within 0.05 km, 0.05 km-1 km, 1 km-5 km, and 5–10 km (*F*=3.08, *df1*=3, *df*2=325, *p* = .002). No significant relationship was found between these periods and daily traffic crime rate.
