# Supplementary material for: Effect of pachinko parlour openings and closings on neighbourhood income-generating crimes in Japan: 6.5 years of observations
Source: BMC Public Health. 2024 Jul 16;24:1905. doi: 10.1186/s12889-024-19373-1 (PMC11250958; doi:10.1186/s12889-024-19373-1)
Supplement: Supplementary file 9 — Supplementary Material 9. [file 12889_2024_19373_MOESM9_ESM.docx]

Additional file 9. Effect of opening a pachinko parlour on income-generating and traffic crime

| Offence | Income-generating crime | | | | | | | | Traffic crime | | | | | | | |
| --- | --- | --- | --- | --- | --- | --- | --- | --- | --- | --- | --- | --- | --- | --- | --- | --- |
| Area | Within 0.5 km | | Within 0.5–1 km | | Within 1–5 km | | Within 5–10 km | | Within 0.5 km | | Within 0.5–1 km | | Within 1–5 km | | Within 5–10 km | |
| Group effect | -0.63 |  | -0.03 |  | 0.05 |  | -0.01 |  | -0.30 |  | 0.07 |  | -0.02 |  | 0.00 |  |
| Time effect | -0.48 |  | -0.07 |  | -0.02 |  | 0.02 |  | -0.07 |  | 0.01 |  | 0.00 |  | 0.00 |  |
| Group×Time effect | 0.71 |  | 0.25 |  | 0.19 | ** | 0.12 | * | 0.04 |  | 0.05 |  | 0.01 |  | 0.00 |  |
| Num. Conv. Effect | 0.26 | ** | 0.10 | ** | -0.07 | ** | -0.05 | ** | 0.06 | ** | 0.01 |  | 0.00 | ** | 0.00 |  |
| Num. Always. Effect | -0.11 |  | 0.22 | ** | 0.31 | ** | 0.19 | ** | -0.01 |  | 0.15 | ** | 0.01 | ** | 0.01 | ** |
| R^2^ | 0.07 |  | 0.16 |  | 0.48 |  | 0.56 |  | 0.03 |  | 0.05 |  | 0.32 |  | 0.48 |  |
| Adj. R^2^ | 0.07 |  | 0.15 |  | 0.48 |  | 0.56 |  | 0.02 |  | 0.04 |  | 0.32 |  | 0.48 |  |

*Notes.* Num. Conv.: Number of convenience stores within 5 km. Num. Always.: Number of always open pachinko parlors in the neighborhood. *: *p* < .05, **: *p* < .01
