# Supplementary material for: Effect of pachinko parlour openings and closings on neighbourhood income-generating crimes in Japan: 6.5 years of observations
Source: BMC Public Health. 2024 Jul 16;24:1905. doi: 10.1186/s12889-024-19373-1 (PMC11250958; doi:10.1186/s12889-024-19373-1)
Supplement: Supplementary file 11 — Supplementary Material 11. [file 12889_2024_19373_MOESM11_ESM.docx]

Additional file 11. Effect of pachinko parlour opening on purse snatch and theft

| Offence | Purse snatch | | | | | | | | Theft | | | | | | | |
| --- | --- | --- | --- | --- | --- | --- | --- | --- | --- | --- | --- | --- | --- | --- | --- | --- |
| Area | Within 0.5 km | | Within 0.5–1 km | | Within 1–5 km | | Within 5–10 km | | Within 0.5 km | | Within 0.5–1 km | | Within 1–5 km | | Within 5–10 km | |
| Group effect | -0.13 | * | -0.04 |  | 0.00 |  | -0.01 |  | -0.52 | * | -0.02 |  | -0.01 |  | 0.00 |  |
| Time effect | -0.14 | ** | -0.08 | ** | -0.03 | ** | -0.02 | ** | -0.20 |  | -0.04 |  | -0.03 | ** | -0.01 | ** |
| Group×Time effect | 0.09 |  | 0.02 |  | 0.00 |  | 0.00 |  | 0.16 |  | -0.02 |  | 0.02 |  | 0.00 |  |
| Num. Conv. Effect | 0.02 | ** | 0.01 | ** | -0.01 | ** | 0.00 | ** | 0.10 | ** | 0.03 | ** | 0.01 | * | 0.00 | ** |
| Num. Always. Effect | 0.20 | ** | 0.09 | ** | 0.03 | ** | 0.01 | ** | 0.00 |  | 0.03 |  | 0.02 | ** | 0.02 | ** |
| R^2^ | 0.24 |  | 0.17 |  | 0.34 |  | 0.36 |  | 0.05 |  | 0.06 |  | 0.30 |  | 0.44 |  |
| Adj. R^2^ | 0.24 |  | 0.17 |  | 0.34 |  | 0.36 |  | 0.05 |  | 0.06 |  | 0.29 |  | 0.43 |  |

Notes. Num. Conv.: Number of convenience stores within 5 km. Num. Always.: Number of always open pachinko parlors in the neighborhood. *: *p* < .05, **: *p* < .01
