# Supplementary material for: Effect of pachinko parlour openings and closings on neighbourhood income-generating crimes in Japan: 6.5 years of observations
Source: BMC Public Health. 2024 Jul 16;24:1905. doi: 10.1186/s12889-024-19373-1 (PMC11250958; doi:10.1186/s12889-024-19373-1)
Supplement: Supplementary file 12 — Supplementary Material 12. [file 12889_2024_19373_MOESM12_ESM.docx]

Additional file 12. Effect of pachinko parlour closing on income-generating and traffic crimes

| Offence | Income-generating crime | | | | | | | | Traffic crime | | | | | | | |
| --- | --- | --- | --- | --- | --- | --- | --- | --- | --- | --- | --- | --- | --- | --- | --- | --- |
| Area | Within 0.5 km | | Within 0.5–1 km | | Within 1–5 km | | Within 5–10 km | | Within 0.5 km | | Within 0.5–1 km | | Within 1–5 km | | Within 5–10 km | |
| Group effect | 0.31 | * | -0.47 | ** | -0.21 | ** | -0.16 | ** | 0.11 | ** | 0.00 |  | -0.01 | ** | 0.00 | * |
| Time effect | -0.74 | ** | -0.30 | ** | 0.03 |  | 0.02 |  | -0.06 | * | -0.01 |  | -0.01 | ** | 0.00 | ** |
| Group×Time effect | 0.93 | ** | 0.62 | ** | 0.22 | ** | 0.17 | ** | 0.02 |  | 0.03 |  | 0.01 | ** | 0.00 |  |
| Num. Conv. Effect | 0.10 | ** | 0.17 | ** | -0.09 | ** | -0.06 | ** | 0.02 | ** | 0.01 | ** | 0.01 | ** | 0.00 |  |
| Num. Always. Effect | 1.19 | ** | 0.36 | ** | 0.46 | ** | 0.27 | ** | 0.07 | ** | 0.04 | ** | 0.01 | ** | 0.01 | ** |
| R^2^ | 0.12 |  | 0.21 |  | 0.53 |  | 0.60 |  | 0.03 |  | 0.07 |  | 0.33 |  | 0.53 |  |
| Adj. R^2^ | 0.12 |  | 0.21 |  | 0.53 |  | 0.60 |  | 0.03 |  | 0.07 |  | 0.33 |  | 0.53 |  |

*Notes.* Num. Conv.: Number of convenience stores within 5 km. Num. Always.: Number of always open pachinko parlors in the neighborhood. *: *p* < .05, **: *p* < .01
