# Supplementary material for: Effect of pachinko parlour openings and closings on neighbourhood income-generating crimes in Japan: 6.5 years of observations
Source: BMC Public Health. 2024 Jul 16;24:1905. doi: 10.1186/s12889-024-19373-1 (PMC11250958; doi:10.1186/s12889-024-19373-1)
Supplement: Supplementary file 13 — Supplementary Material 13. [file 12889_2024_19373_MOESM13_ESM.docx]

Additional file 13. Effect of pachinko parlour closing on fraud and robbery

| Offence | Fraud | | | | | | | | Robbery | | | | | | | |
| --- | --- | --- | --- | --- | --- | --- | --- | --- | --- | --- | --- | --- | --- | --- | --- | --- |
| Area | Within 0.5 km | | Within 0.5–1 km | | Within 1–5 km | | Within 5–10 km | | Within 0.5 km | | Within 0.5–1 km | | Within 1–5 km | | Within 5–10 km | |
| Group effect | 0.18 |  | -0.45 | ** | -0.20 | ** | -0.15 | ** | 0.04 | ** | -0.02 | ** | 0.00 | ** | 0.00 | ** |
| Time effect | -0.47 | ** | -0.17 | ** | 0.08 | ** | 0.05 | ** | -0.02 | * | -0.02 | ** | 0.00 | ** | 0.00 | ** |
| Group×Time effect | 0.83 | ** | 0.55 | ** | 0.21 | ** | 0.17 | ** | 0.01 |  | 0.02 | * | 0.00 | ** | 0.00 | ** |
| Num. Conv. Effect | 0.08 | ** | 0.14 | ** | -0.09 | ** | -0.06 | ** | 0.00 |  | 0.01 | ** | 0.00 | ** | 0.00 | ** |
| Num. Always. Effect | 1.06 | ** | 0.26 | ** | 0.42 | ** | 0.25 | ** | 0.06 | ** | 0.02 | ** | 0.01 | ** | 0.01 | ** |
| R^2^ | 0.11 |  | 0.19 |  | 0.49 |  | 0.55 |  | 0.07 |  | 0.04 |  | 0.39 |  | 0.53 |  |
| Adj. R^2^ | 0.11 |  | 0.19 |  | 0.49 |  | 0.55 |  | 0.07 |  | 0.04 |  | 0.39 |  | 0.53 |  |

*Notes.* Num. Conv.: Number of convenience stores within 5 km. Num. Always.: Number of always open pachinko parlors in the neighborhood. *: *p* < .05, **: *p* < .01
