# Supplementary material for: Effect of pachinko parlour openings and closings on neighbourhood income-generating crimes in Japan: 6.5 years of observations
Source: BMC Public Health. 2024 Jul 16;24:1905. doi: 10.1186/s12889-024-19373-1 (PMC11250958; doi:10.1186/s12889-024-19373-1)
Supplement: Supplementary file 15 — Supplementary Material 15. [file 12889_2024_19373_MOESM15_ESM.docx]

Additional file 15. Additional values of Table 5

|  |  |  |  |  | Daily income-generating crime rate | | | | | | |
| --- | --- | --- | --- | --- | --- | --- | --- | --- | --- | --- | --- |
| Distance | Type |  | Num. conv. | Num. always. | 2017  (365 days) | 2018  (365 days) | 2019  (365 days) | 2020  (366 days) | 2021  (365 days) | 2022  (365 days) | 2023  (153 days) |
| Within 0.5 km | always closed. | Min | 0 | 0 | 0.00 | 0.00 | 0.00 | 0.00 | 0.00 | 0.00 | 0.00 |
|  | (n=4430) | Max | 1796 | 11 | 81.98 | 72.91 | 90.35 | 85.46 | 78.49 | 89.30 | 98.20 |
|  | opened-then closed. | Min | 0 | 0 | 0.00 | 0.00 | 0.00 | 0.00 | 0.00 | 0.00 | 0.00 |
|  | (n=3593) | Max | 1814 | 11 | 65.93 | 66.28 | 68.02 | 79.53 | 76.39 | 138.84 | 88.21 |
|  | always open. | Min | 0 | 1 | 0.00 | 0.00 | 0.00 | 0.00 | 0.00 | 0.00 | 0.00 |
|  | (n=3549) | Max | 1783 | 11 | 96.63 | 90.70 | 65.58 | 79.53 | 76.39 | 60.35 | 56.59 |
| Within 0.5–1 km | always closed. | Min | 0 | 0 | 0.00 | 0.00 | 0.00 | 0.00 | 0.00 | 0.00 | 0.00 |
|  | (n=4430) | Max | 1796 | 13 | 32.21 | 30.23 | 30.23 | 28.84 | 29.53 | 46.51 | 53.26 |
|  | opened-then closed. | Min | 0 | 0 | 0.00 | 0.00 | 0.00 | 0.00 | 0.00 | 0.00 | 0.00 |
|  | (n=3593) | Max | 1814 | 10 | 27.44 | 18.60 | 30.23 | 28.49 | 27.44 | 29.65 | 52.70 |
|  | always open. | Min | 0 | 0 | 0.00 | 0.00 | 0.00 | 0.00 | 0.00 | 0.00 | 0.00 |
|  | (n=3549) | Max | 1783 | 13 | 27.91 | 24.30 | 24.19 | 27.91 | 26.16 | 29.77 | 52.70 |
| Within 1–5 km | always closed. | Min | 0 | 0 | 0.00 | 0.00 | 0.00 | 0.00 | 0.00 | 0.00 | 0.00 |
|  | (n=4430) | Max | 1796 | 78 | 3.47 | 3.92 | 4.73 | 5.61 | 7.30 | 7.12 | 5.99 |
|  | opened-then closed. | Min | 0 | 0 | 0.00 | 0.00 | 0.00 | 0.00 | 0.00 | 0.00 | 0.00 |
|  | (n=3593) | Max | 1814 | 74 | 3.32 | 3.62 | 4.67 | 5.35 | 7.14 | 7.19 | 5.87 |
|  | always open. | Min | 0 | 0 | 0.00 | 0.00 | 0.00 | 0.00 | 0.00 | 0.00 | 0.00 |
|  | (n=3549) | Max | 1783 | 68 | 3.34 | 3.84 | 4.59 | 5.38 | 7.14 | 7.15 | 5.96 |
| Within 5–10 km | always closed. | Min | 0 | 0 | 0.00 | 0.00 | 0.00 | 0.00 | 0.00 | 0.00 | 0.00 |
|  | (n=4430) | Max | 1796 | 145 | 2.06 | 1.98 | 2.22 | 3.72 | 5.76 | 5.83 | 4.11 |
|  | opened-then closed. | Min | 0 | 0 | 0.00 | 0.00 | 0.00 | 0.00 | 0.00 | 0.00 | 0.00 |
|  | (n=3593) | Max | 1814 | 144 | 2.05 | 1.93 | 2.21 | 3.58 | 5.47 | 5.58 | 4.06 |
|  | always open. | Min | 0 | 0 | 0.00 | 0.00 | 0.00 | 0.00 | 0.00 | 0.00 | 0.00 |
|  | (n=3549) | Max | 1783 | 147 | 2.06 | 1.96 | 2.18 | 3.70 | 5.73 | 5.82 | 4.06 |

*Notes.* Num. Conv.: Number of convenience stores within 5 km. Num. Always.: Number of always open pachinko parlors in the neighborhood.

The daily income-generating crime rates in the neighborhood of the pachinko parlors were significantly associated with the number of convenience stores in the neighborhood of the pachinko parlors(*F*= 14616.62, *df1*=1, *df2*=324002, *p* < .001), but not the number of always open pachinko parlors open (*F*= 0.89, *df1*=1, *df2*=324002, *p* = .345).
