# Supplementary material for: Effect of pachinko parlour openings and closings on neighbourhood income-generating crimes in Japan: 6.5 years of observations
Source: BMC Public Health. 2024 Jul 16;24:1905. doi: 10.1186/s12889-024-19373-1 (PMC11250958; doi:10.1186/s12889-024-19373-1)
Supplement: Supplementary file 16 — Supplementary Material 16. [file 12889_2024_19373_MOESM16_ESM.docx]

Additional file 16. Comparison of 6.5-year traffic crime rates by pachinko parlour types

|  |  |  |  |  | Daily income-generating crime rate | | | | | | |
| --- | --- | --- | --- | --- | --- | --- | --- | --- | --- | --- | --- |
| Distance | Type |  | Num. conv. | Num. always. | 2017  (365 days) | 2018  (365 days) | 2019  (365 days) | 2020  (366 days) | 2021  (365 days) | 2022  (365 days) | 2023  (153 days) |
| Within 0.5 km | always closed. | M | 213.10 | 0.58 | 0.20 | 0.39 | 0.28 | 0.21 | 0.24 | 0.25 | 0.20 |
|  | (n=4430) | S.D. | 316.40 | 1.20 | 1.44 | 1.92 | 2.00 | 1.64 | 1.55 | 1.69 | 1.26 |
|  | opened-then closed. | M | 187.00 | 0.45 | 0.17 | 0.31 | 0.22 | 0.18 | 0.18 | 0.20 | 0.14 |
|  | (n=3593) | S.D. | 266.02 | 1.02 | 1.43 | 1.83 | 1.74 | 1.33 | 1.36 | 1.52 | 1.02 |
|  | always open. | M | 182.80 | 1.44 | 0.12 | 0.23 | 0.14 | 0.11 | 0.12 | 0.15 | 0.10 |
|  | (n=3549) | S.D. | 251.84 | 1.04 | 0.99 | 1.36 | 1.11 | 0.80 | 0.91 | 1.05 | 0.71 |
| Within 0.5–1 km | always closed. | M | 213.10 | 0.47 | 0.11 | 0.21 | 0.15 | 0.13 | 0.14 | 0.15 | 0.12 |
|  | (n=4430) | S.D. | 316.40 | 0.97 | 0.45 | 0.74 | 0.82 | 0.74 | 0.78 | 0.79 | 0.61 |
|  | opened-then closed. | M | 187.00 | 0.39 | 0.08 | 0.14 | 0.12 | 0.10 | 0.11 | 0.13 | 0.10 |
|  | (n=3593) | S.D. | 266.02 | 0.81 | 0.34 | 0.53 | 0.60 | 0.54 | 0.60 | 0.71 | 0.55 |
|  | always open. | M | 182.80 | 0.39 | 0.08 | 0.12 | 0.09 | 0.08 | 0.09 | 0.10 | 0.07 |
|  | (n=3549) | S.D. | 251.84 | 0.80 | 0.34 | 0.53 | 0.36 | 0.30 | 0.35 | 0.50 | 0.34 |
| Within 1–5 km | always closed. | M | 213.10 | 9.90 | 0.04 | 0.10 | 0.06 | 0.05 | 0.05 | 0.06 | 0.05 |
|  | (n=4430) | S.D. | 316.40 | 12.38 | 0.07 | 0.20 | 0.11 | 0.09 | 0.10 | 0.11 | 0.09 |
|  | opened-then closed. | M | 187.00 | 9.35 | 0.04 | 0.08 | 0.05 | 0.04 | 0.05 | 0.06 | 0.05 |
|  | (n=3593) | S.D. | 266.02 | 11.56 | 0.07 | 0.16 | 0.10 | 0.08 | 0.09 | 0.11 | 0.09 |
|  | always open. | M | 182.80 | 9.34 | 0.04 | 0.08 | 0.05 | 0.04 | 0.05 | 0.06 | 0.05 |
|  | (n=3549) | S.D. | 251.84 | 11.84 | 0.07 | 0.14 | 0.10 | 0.08 | 0.09 | 0.10 | 0.08 |
| Within 5–10 km | always closed. | M | 213.10 | 22.11 | 0.02 | 0.06 | 0.03 | 0.03 | 0.03 | 0.03 | 0.03 |
|  | (n=4430) | S.D. | 316.40 | 29.39 | 0.03 | 0.10 | 0.05 | 0.04 | 0.04 | 0.05 | 0.04 |
|  | opened-then closed. | M | 187.00 | 21.24 | 0.02 | 0.06 | 0.03 | 0.03 | 0.03 | 0.03 | 0.03 |
|  | (n=3593) | S.D. | 266.02 | 28.00 | 0.03 | 0.09 | 0.05 | 0.04 | 0.04 | 0.05 | 0.04 |
|  | always open. | M | 182.80 | 21.78 | 0.03 | 0.05 | 0.03 | 0.03 | 0.03 | 0.03 | 0.03 |
|  | (n=3549) | S.D. | 251.84 | 28.80 | 0.03 | 0.09 | 0.05 | 0.04 | 0.04 | 0.05 | 0.04 |

*Notes.* Num. Conv.: Number of convenience stores within 5 km. Num. Always.: Number of always open pachinko parlors in the neighborhood.

Traffic crime rates were significantly different among the pachinko parlour types (*F*=82.21, *df1*=2, *df2*=324002, *p* < .001). Significant between-group differences were also found in the distances from pachinko parlours (*F*=349.95, *df1*=3, *df2*=324002, *p* < .001) and years (*F*=51.77, *df1*=6, *df2*=324002, *p* < .001).

Multiple comparisons also showed that traffic crime rates of closed pachinko parlours were significantly higher than those of open and always open pachinko parlours. Furthermore, the number of opened then closed pachinko parlours was significantly higher than that of always open pachinko parlours.
