# Supplementary material for: Effect of pachinko parlour openings and closings on neighbourhood income-generating crimes in Japan: 6.5 years of observations
Source: BMC Public Health. 2024 Jul 16;24:1905. doi: 10.1186/s12889-024-19373-1 (PMC11250958; doi:10.1186/s12889-024-19373-1)
Supplement: Supplementary file 17 — Supplementary Material 17. [file 12889_2024_19373_MOESM17_ESM.docx]

Additional file 17. Comparison of daily traffic crime rates between 2017 and 2023 among pachinko parlours in Japan

| Area within 0.5 km of pachinko parlours | Area within 0.5 km to 1 km of pachinko parlours |
| --- | --- |
| 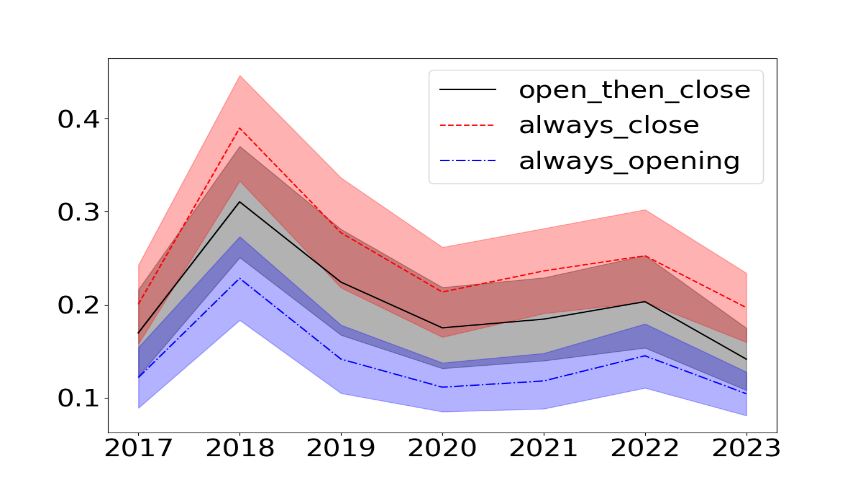  Daily traffic crime rate  Years  Years | 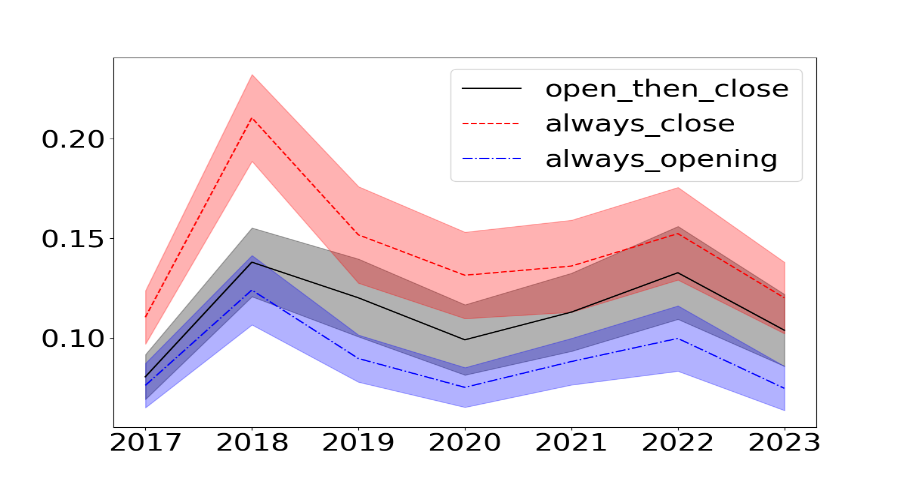  Daily traffic crime rate  Years |
| Area within 1 km to 5 km of pachinko parlours | Area within 5 km to 10 km of pachinko parlours |
| 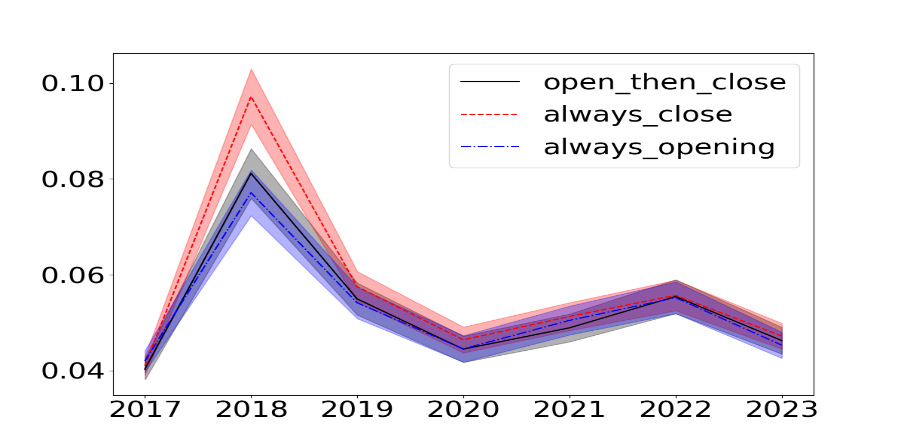  Daily traffic crime rate  Years | 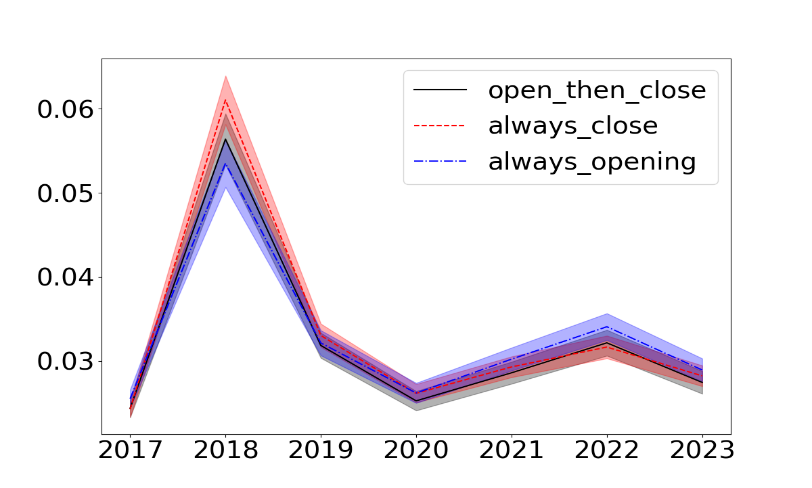  Daily traffic crime rate  Years |

n = 11,572 pachinko parlours× 7 years

*Notes.* Lightly painted areas indicate 95% confidence intervals. Traffic crime rates were significantly different among the pachinko parlour types (*F*=82.21, *df1*=2, *df2*=324002, *p* < .001). Significant between-group differences were also found in the distances from pachinko parlours (*F*=349.95, *df1*=3, *df2*=324002, *p* < .001) and years (*F*=51.77, *df1*=6, *df2*=324002, *p* < .001).

Multiple comparisons also showed that traffic crime rates of closed pachinko parlours were significantly higher than those of open and always open pachinko parlours. Furthermore, the number of opened then closed pachinko parlours was significantly higher than that of always open pachinko parlours.
